# Astrocyte store released calcium modulates structural synapse development and circuit output in the visual system

**DOI:** 10.1038/s42003-026-10579-0

**Published:** 2026-07-11

**Authors:** Gillian Imrie, Jordan Mar, Madison B. Gray, Tatiana Quiceno, Isabella Farhy-Tselnicker

**Affiliations:** 1 Department of Biology, Texas A&M University, College Station, TX USA; 2 Texas A&M Institute for Neuroscience (TAMIN), Texas A&M University, College Station, TX USA; 3 Center for Biological Clocks Research, Texas A&M University, College Station, TX USA

**Keywords:** Astrocyte, Synaptic development

## Abstract

Astrocytes, a major class of glial cells, are critical regulators of neuronal synapse development and function. Deficits in astrocyte-synapse interactions are implicated in various neurological disorders, yet the precise mechanisms by which astrocytes guide synapse formation and maturation during development remain poorly understood. A key astrocytic mechanism for integrating both extrinsic (e.g., neuronal) and intrinsic signals is the IP3-mediated release of intracellular calcium (Ca^2+^) from endoplasmic reticulum (ER) stores, modulating a wide range of downstream effects. Though defects in this signaling pathway have been linked to adult brain dysfunction, its role in shaping synaptic development, a period when astrocyte-neuron communication is established, is largely unknown. Here, we investigate the role of IP3-mediated Ca^2+^ signaling in synapse development in the mouse visual system. Using a combination of histological, molecular, and circuit-level approaches, we find that loss of the IP3 receptor (IP3R type 2; IP3R2) leads to significant deficits in the structural maturation of glutamatergic synapses. These synaptic disruptions are accompanied by attenuated visually evoked neuronal activation and impaired behavioral responses to visual threat stimuli. We further show that astrocytic morphological complexity is diminished in the absence of IP3R2, suggesting that store-released Ca^2+^ is required for the maturation of astrocyte-neuron interactions. Our findings establish a critical role for IP3R2-mediated Ca^2+^ signaling in shaping excitatory circuit development and the emergence of visually driven behaviors.

## Abstract (web summary)

Disruption of astrocytic IP3R2-mediated calcium signaling demonstrates its critical role in structural excitatory synapse maturation, astrocyte morphological complexity, and behavioral output within the developing visual system.

## Introduction

Synapses, the fundamental units of electrochemical communication in the brain, are tightly regulated by astrocyte activity throughout all life stages^1–3^. During early postnatal development, when excitatory and inhibitory neuronal circuits are actively forming and refining, astrocytic regulatory functions influence the maturation, strength, and specificity of synaptic connections^2,4,5^. Dysregulation of synapse formation is linked to numerous brain pathologies, including depression and mood disorders^6^, autism spectrum disorders^7^, and epilepsy^8^; however, the precise mechanisms by which astrocytes regulate synapse formation and maturation are not yet fully understood.

In the mouse visual cortex (VC), synapse development and maturation follows a well-defined postnatal trajectory starting at around postnatal day (P) 7, peaking at P14 concurrently with eye opening, and stabilizing near P28^2^. Astrocyte development is temporally aligned with these synaptogenic stages^2,9,10^, during which astrocytes engage in robust bidirectional communication with neurons and undergo major changes in gene expression and structural morphology which promote the appropriate spatiotemporal recruitment of synaptic components in a neuronal activity-dependent manner^4,11^. This allows astrocytes to dynamically signal in a rapidly changing environment and to integrate neuronal activity in ways appropriate to developmental stage and context^4,11,12^. Though the importance of these developmental interactions has been described in previous work^2–5,13^, the exact signaling pathways activated in astrocytes to modulate them remain unresolved.

A central mechanism underlying astrocytic responses to intrinsic and extrinsic cues occurs via fluctuations in intracellular calcium (Ca^2+^). Astrocytic Ca^2+^ signals arise from diverse sources, exhibit complex spatial and temporal dynamics, and have been associated with multiple regulatory functions from the cellular to behavioral levels^14–17^, including energy metabolism, neuronal circuit synchronization, network scaling, and encoding of sensory stimulus details like salience and context^18–23^. A major pathway by which astrocytes integrate neuronal signals involves Ca^2+^ release from endoplasmic reticulum (ER) stores^24^. Neurotransmitters and neuromodulators bind to astrocytic G-protein coupled receptors (GPCRs), activating phospholipase C to generate inositol 1,4,5-trisphosphate (IP3). IP3 binds to its receptors on the ER, which in cortical astrocytes are predominantly IP3 receptors type 2 (IP3R2)^25–29^, triggering Ca^2+^ release into the cytosol modulating various downstream effects^12,30–37^. The physiological role of IP3R2-mediated Ca^2+^ signaling remains somewhat controversial, as while some studies link its disruption to functional deficits like cortical dysregulation and autism-like behaviors^4,17,22,31,37,38^, others report no effects^26,39^. This conflicting evidence likely stems from the use of variant knockout strategies, developmental stages, and diverse brain regions across different studies. Importantly, most of the work investigating IP3R2-mediated signaling has been focused on adults, while little is known about the involvement of this signaling pathway in modulating synapses during development, a time when astrocyte-neuronal communication is established. Thus, thorough investigation into the brain region-, circuit-, and developmental stage-specific functions of this signaling pathway is needed.

Here, we determine the role of IP3R2-mediated store-released Ca^2+^ signaling in the astrocyte-dependent regulation of synapse development within the mouse visual circuit. Using a combination of histological, molecular, and circuit level approaches across defined postnatal timepoints, we demonstrate that loss of IP3R2 leads to deficits in the structural maturation of glutamatergic synapses in the primary visual cortex (VC). These synaptic disruptions are accompanied by attenuated visually evoked neuronal activation across multiple visual circuit-relevant brain regions, and impaired behavioral responses to visual threat stimuli. We further show that astrocyte morphological complexity in the VC is diminished in the absence of IP3R2 at P16 and P30, when astrocytic synapse regulation and processing of visual information following eye opening peaks and stabilizes, suggesting that store-released Ca^2+^ is required for the maturation of astrocyte-neuron interactions. Our findings establish a critical role for IP3R2-mediated Ca^2+^ signaling in shaping the development of excitatory circuits and visually driven behaviors.

## Results

**Fig. 1:**
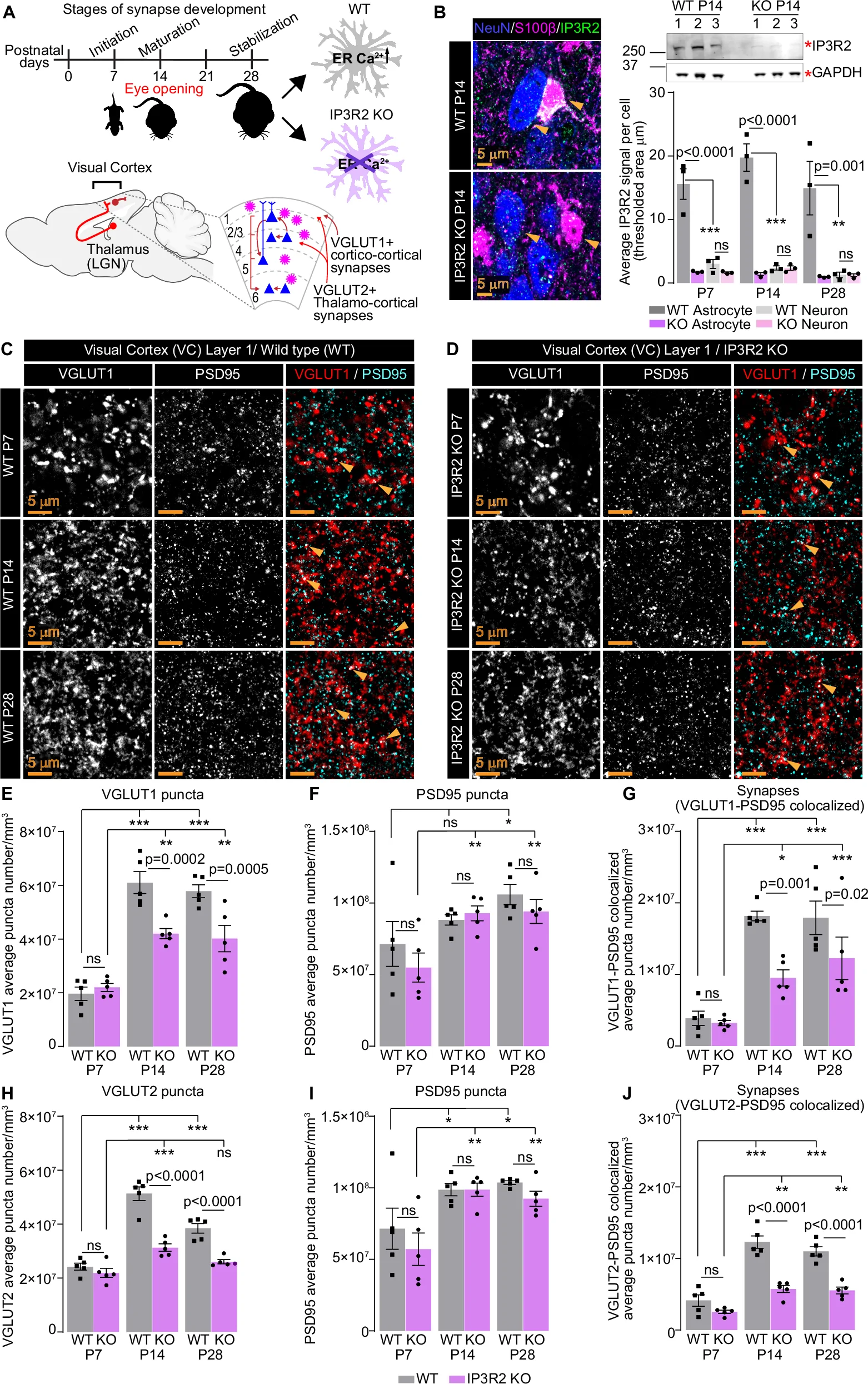
Glutamatergic synapse development is perturbed in IP3R2 KO mice VC. See also Figure S1, S2, Supplementary Data 1. **A** Schematic of experiment: Brain tissue is collected from WT and KO mice at P7, P14 and P28 corresponding to stages of synapse development (top panel); IHC to quantify synapses as indicated is performed in Layer 1 of the VC for VGLUT1-containing cortico-cortical synapses, and VGLUT2-containing thalamo-cortical synapses (bottom panel). **B** Validation of IP3R2 KO by IHC and WB. Left panel - example high power (×63 objective) confocal images of IP3R2 (green), astrocyte marker S100β (magenta) and neuronal marker Neun (blue) in the VC at P14 (see also Fig. S1A-B). IP3R2 colocalized with the S100β signal appears as white color. Graph is the quantification of colocalized signal with each cell marker at P7, P14, and P28 as labeled. IP3R2 signal is highly colocalized with astrocytes and not with neurons and is downregulated in KO VC. Top panel is example WB image showing IP3R2 band ( ~ 250KDa) and GAPDH (loading control, ~36 KDa) in WT and KO at P14 as labeled. Numbers indicate samples from individual animals. Uncropped blots are shown in Fig. S1G–I. **C–G** VGLUT1-containing synapses are reduced in IP3R2 KO VC at P14 and P28 but not at P7. Example images of the presynaptic VGLUT1, postsynaptic PSD95 and merged (synapses) in each age and genotype as labeled (**C**,** D**), and quantification of individual synaptic proteins and synapse number per mm^3^ represented as colocalization between VGLUT1 and PSD95 for both genotypes (**E**,** G**) are shown. Single channel grayscale images on the left, merged images on the right. The number of VGLUT1 puncta and VGLUT1-containing synapses is reduced in IP3R2 KO (**E**,** G**), while PSD95 numbers are unaltered (**F**). **H–J** VGLUT2-containing synapses are reduced in IP3R2 KO VC at P14 and P28 but not at P7. Quantification of individual synaptic proteins and synapse number per mm^3^ represented as colocalization between VGLUT2 and PSD95 for both genotypes are shown (see also Fig. S2A–B). The number of VGLUT2 puncta and VGLUT2-containing synapses is reduced in KO (**H**,** J**), while PSD95 numbers are unaltered (**I**). Graphs show mean ± s.e.m. Individual mouse data points are shown as circles and squares. Number of mice/group (N) *N* = 3 in (**B**); *N* = 5 in (**E**–**J**). Scale bar = 5 μm. Arrowheads mark representative IP3R2 signal colocalized with astrocyte marker S100β or neuronal marker Neun in (**B**) and colocalized synaptic puncta in (**C**, **D**). Statistical analysis by two-way ANOVA with multiple comparison correction by the two-stage linear step-up procedure of Benjamini, Kriger and Yekutieli examining the effects of cell type and genotype (**B**), and age and genotype (**E**–**J**). *P ≤ 0.05; **P < 0.01; ***P < 0.001 comparing age/cell type groups within each genotype; *P* value (p) on the plots comparing WT and KO groups. ns denotes non-significant results (*P* > 0.05). Descriptive statistics and detailed statistical test results for all data panels are provided in Supplementary Data 1.

Astrocytic intracellular Ca^2+^ fluctuations are fundamental to their regulatory roles at the neuronal synapse. While these Ca^2+^signals are characterized by a diversity of forms and origins^40,41^, store-released Ca^2+^ mediated by IP3R2 couples synaptic neurotransmitter release to changes in synapse regulating genetic programs in astrocytes^4,42^. We previously identified that mice lacking IP3R2, which is essential for astrocytic store-released Ca^2+^ signaling^31,39^, have dysregulated expression of synapse modulating genes including Glypican 4 and Chordin like 1, and decreased expression levels of vesicular glutamate transporters at P14^4^. To investigate whether synapses in IP3R2 knockout (KO) mice are broadly dysregulated across development, we profiled the expression of pre- and post-synaptic proteins in layer 1 of the visual cortex (VC) of wild-type (WT) and IP3R2 KO mice at P7, P14, and P28 which correspond to cortical synaptogenic onset, peak, and stabilization respectively^2^ (Fig. 1A). We first confirmed the astrocytic expression of IP3R2 and its deletion in the germline KO mice at the mRNA and protein levels using real-time quantitative PCR (qPCR), immunohistochemistry (IHC), and western blot (WB) across these developmental stages (P7, P14, P28) (Fig. 1B, Fig. S1). IP3R2 mRNA (gene name *Itpr2*) was undetectable in IP3R2 KO mice VC (shown as mean ± s.e.m. [ΔCt relative to *Gapdh*]: WT 5.09 ± 0.16; KO 0.00 ± 0.00; Fig. S1F). For IHC, brain sections were co-labeled with astrocyte specific marker S100β and neuronal specific marker Neun to determine cell-type specificity of IP3R2 expression. Consistent with previous studies^25,27–29^, our results show IP3R2 signal exhibiting strong somatic labeling as well as weaker, diffuse signal, which predominantly colocalized with S100β but not with Neun, suggesting astrocytes as the major source of this receptor within the developing VC gray matter. Expression levels of IP3R2 were stable across developmental stages and cortical layers (Fig. 1B, Fig. S1A-B, D-E). Furthermore, we observed a strong reduction of total (Fig. S1C, G–I) and astrocyte specific (S100β colocalized; Fig. 1B, Fig. S1D-E) IP3R2 protein in KO VC, confirming this model’s validity for our experiments (IHC data in Fig. 1B, Fig. S1A-B shown as mean ± s.e.m. [thresholded area µm^2^]: IP3R2-S100β colocalized [P7] WT 15.58 ± 2.4, KO 1.76 ± 0.08; [P14] WT 19.75 ± 2.15, KO 1.41 ± 0.24; [P28] WT 14.94 ± 4.21, KO 0.97 ± 0.07; IP3R2-Neun colocalized [P7] WT 3.006 ± 0.71, KO 1.65 ± 0.12; [P14] WT 2.37 ± 0.33, KO 2.34 ± 0.34; [P28] WT 1.28 ± 0.43, KO 1.22 ± 0.23; IHC data in Fig. S1C shown as mean ± s.e.m. [thresholded area of signal within ROI 101.4 µm^2^]: [P7] WT 181.9 ± 27.33, KO 90.49 ± 3.75; [P14] WT 217.2 ± 23.44, KO 119.9 ± 22.32; [28] WT 135.3 ± 40.17, KO 56.22 ± 2.74; Background signal 38.45 ± 9.63 when primary antibody is omitted (secondary Ab control). WB data is shown as mean ± s.e.m normalized to GAPDH: [P7] WT 0.13 ± 0.007, KO 0.002 ± 0.001; [P14] WT 0.15 ± 0.01, KO 0.02 ± 0.009; [P28] WT 0.06 ± 0.003, KO: 0.01 ± 0.0005; Fig. 1B, Fig. S1G-I). Notably, IP3R2 labeling by IHC was variable across cortical astrocytes, which may reflect dynamic or heterogeneous expression of this protein within the astrocyte population.

### Vesicular glutamate transporters and synapse numbers are developmentally reduced in the visual cortex of IP3R2 knockout mice

To determine how glutamatergic synapse development is impacted by IP3R2 KO, we used IHC to quantify structural synapses of two glutamatergic circuits within the VC, the presynaptic vesicular glutamate transporter 1 (VGLUT1) specific cortico-cortical circuit and vesicular glutamate transporter 2 (VGLUT2) specific thalamo-cortical circuit together with the excitatory postsynaptic density protein 95 (PSD95). We quantified both the numbers of pre- and postsynaptic puncta and their colocalizations to calculate synapse number across developmental stages^4^ (Fig. 1C–J, Fig. S2A,B). We observed a significant decrease in VGLUT1 ( ~ 30%) and VGLUT2 ( ~ 40%) at P14 (in accordance with our previous work)^4^ which persisted to P28 in VC sections from IP3R2 KO mice ( ~ 30% for both proteins) (data shown as mean ± s.e.m. [puncta number per mm³]: VGLUT1 [P14] WT 6.10E7 ± 4.08E6, KO 4.20E7 ± 1.89E6, [P28] WT 5.78E7 ± 2.40E6, KO 4.02E7 ± 4.92E6; VGLUT2 [P14] WT 4.92E7 ± 2.43E6, KO 2.99E7 ± 1.32E6, [P28] WT 3.68E7 ± 1.72E6, KO 2.49E7 ± 7.76E5; Fig. 1E, H), with no change at P7 (VGLUT1 WT 1.96E7 ± 2.53E6, KO 2.20E7 ± 1.49E6; VGLUT2 WT 2.31E7 ± 1.25E6, KO 2.09E7 ± 1.63E6; Fig. 1E, H). Similarly, synapse numbers were decreased for both VGLUT1 and VGLUT2-containing projections at P14 ( ~ 41% and 35% respectively) and P28 ( ~ 32% and 50% respectively) (VGLUT1-PSD95 colocalized [P14] WT 1.82E7 ± 6.72E5, KO 9.51E6 ± 1.13E6; [P28] WT 1.79E7 ± 2.34E6, KO 1.23E7 ± 2.98E6; VGLUT2-PSD95 colocalized [P14] WT 1.23E7 ± 8.69E5, KO 5.73E6 ± 4.93E5; [P28] WT 1.10E7 ± 6.39E5, KO 5.53E6 ± 4.77E5; Fig.1G, J), with no difference at P7 (VGLUT1-PSD95 colocalized WT 3.87E6 ± 1.01E6, KO 3.22E6 ± 3.71E5; VGLUT2-PSD95 colocalized WT 4.14E6 ± 7.92E5, KO 2.55E6 ± 2.41E5; Fig. 1G, J). The observed synaptic deficits were driven by the reduction in presynaptic proteins, as levels of PSD95 were unchanged between WT and IP3R2 KO mice at all ages tested ([P7] WT (from VGLUT1 data) 7.14E7 ± 1.57E7; (from VGLUT2 data) 7.14E7 ± 1.44E7, KO (from VGLUT1 data) 5.49E7 ± 1.01E7; (from VGLUT2 data) 5.71E7 ± 1.13E7; [P14] WT (from VGLUT1 data) 8.81E7 ± 3.64E6; (from VGLUT2 data) 9.86E7 ± 4.29E6, KO (from VGLUT1 data) 9.28E7 ± 5.20E6; (from VGLUT2 data) 9.87E7 ± 4.59E6; [P28] WT (from VGLUT1 data) 1.06E8 ± 7.09E6; (from VGLUT2 data) 1.04E8 ± 1.25E6, KO (from VGLUT1 data) 9.41E7 ± 8.42E6; (from VGLUT2 data) 9.24E7 ± 5.30E6; Fig. 1F, I).

To further analyze how VGLUTs are disrupted in IP3R2 KO mice we quantified the sizes of individual puncta (represented as total volumes from Imaris 3D rendering, see “Methods”) and computed the cumulative frequency of VGLUT puncta volumes for WT and IP3R2 KO mice at each age (Fig. S2C–H). We observed significantly reduced VGLUT1 and VGLUT2 volumes at P14 (by ~40%, and ~35% respectively) and P28 (by ~14% and ~20%) in IP3R2 KO mice compared to WT (data shown as mean ± s.e.m. [volume µm³]: VGLUT1 [P14] WT 1.12 ± 0.009, KO 0.66 ± 0.006, [P28] WT 1.0 ± 0.01, KO 0.86 ± 0.01; VGLUT2 [P14] WT 0.79 ± 0.008, KO 0.51 ± 0.007; [P28] WT 0.80 ± 0.009, KO 0.64 ± 0.008; Fig. S2D-E, G-H), with no difference at P7 (VGLUT1 WT 0.89 ± 0.01, KO 0.84 ± 0.01; VGLUT2 WT 0.83 ± 0.01 , KO: 0.78 ± 0.01; Fig. S2C, F). These data suggest that IP3R2 mediated Ca^2+^ signaling is necessary for the developmental maintenance, but not initiation, of glutamatergic synapses in the VC.

### Gross axonal density and cell number are unaffected by IP3R2 knockout

**Fig. 2:**
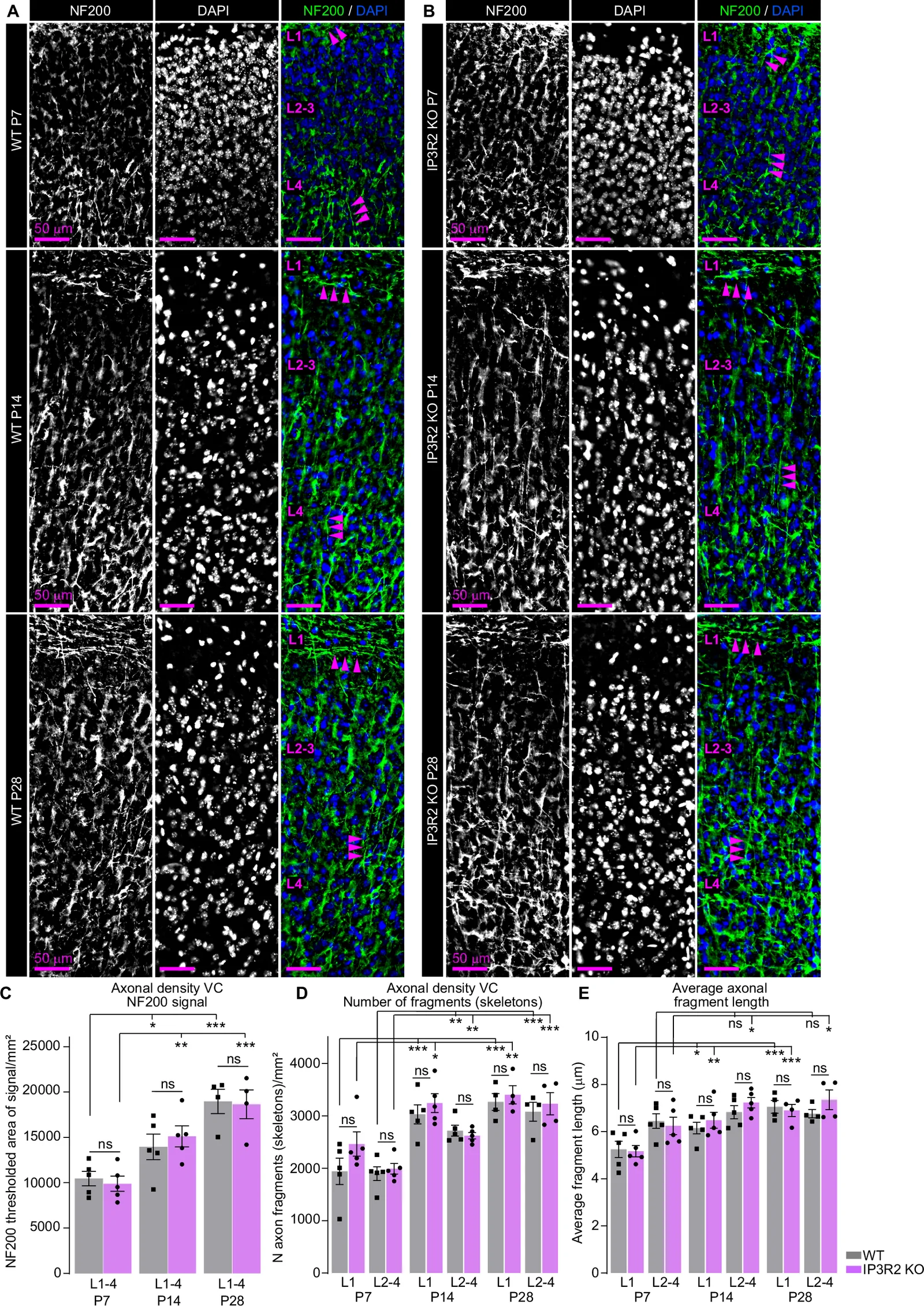
Gross axonal density is unperturbed in IP3R2 KO mice VC. See also Fig. S3, Supplementary Data 1. **A**,** B** Example VC images (layers 1–4) of the axonal marker NF200 (green) and nuclear marker DAPI (blue) in each age and genotype as labeled. Single channel grayscale images on the left, merged images on the right. Quantification of axonal density represented as thresholded area of NF200 fluorescent signal (**C**), number of skeletonized axonal fragments obtained by skeletonize-based analysis (separately analyzed for L1 and L2-4; see “Methods”) (**D**), and average axonal fragment length (from skeletonize-based analysis as in **C**) (**E**) for each age and genotype as labeled. Axonal density is similarly increased in both WT and KO from P7 to P28. No difference between WT and KO is observed at any age, cortical layer, or axonal density parameter analyzed. Graphs show mean ± s.e.m. Individual mouse data points are shown as circles and squares. Number of mice/group (N) *N* = 5. Scale bar = 50 μm. Arrowheads mark representative axons showing different directionality in L1 vs L2-4. Statistical analysis by two-way ANOVA with multiple comparison correction by the two-stage linear step-up procedure of Benjamini, Kriger and Yekutieli examining the effects of age and genotype; *P ≤ 0.05; **P < 0.01; ***P < 0.001 comparing age groups within each genotype; *P* value (p) on the plots comparing WT and KO groups. ns denotes non-significant (*P* > 0.05) results. Descriptive statistics and detailed statistical test results for all data panels are provided in Supplementary Data 1.

The observed decrease in VGLUT1 and VGLUT2 immunoreactivity could be indicative of reduced cortical and thalamic innervation to the VC or overall reduction in cell numbers due to decreased cell survival or proliferation during development. To test this, we immunolabeled VC sections from WT and IP3R2 KO mice at P7, P14, and P28 for the intermediate filament protein neurofilament 200 (NF200)^43^ (Fig. 2A–E). Axonal density, quantified both as changes in NF200 fluorescent signal (Fig. 2C) and skeletonized analysis of axonal fragment number and average length (Fig. 2D, E), increased steadily across developmental stages supporting previous studies^44,45^, with no changes observed between WT and IP3R2 KO mice across any of the timepoints, cortical layers, or analysis parameters. However, subtle differences in axonal branching (including thin or non-myelinated axons) cannot be excluded due to the limitation of NF200 signal which labels primarily large myelinated axons (NF200 signal in Fig. 2C shown as mean ± s.e.m. [thresholded area mm^2^]: [P7] WT 10460 ± 803, KO 9883 ± 836.3, [P14] WT 13953 ± 1414, KO 15108 ± 1163, [P28] WT 20239 ± 1646, KO 18117 ± 1339; skeleton density in Fig. 2D shown as mean ± s.e.m. [number of fragments per mm^2^] [P7] L1 WT 1942 ± 250.4, KO 2462 ± 231.7, L2-4 WT 1897 ± 130.6, KO 1989 ± 102.4; [P14] L1 WT 3030 ± 183.1, KO 3244 ± 180.1, L2-4 WT 2718 ± 102.1, KO 2622 ± 60.78; [P28] L1 WT 3265 ± 161.6, KO 3403 ± 175.5, L2-4 3079 ± 182, KO 3232 ± 210.7. Average axonal fragment length in Fig. 2E shown as mean ± s.e.m. [µm] [P7] L1 WT 5.24 ± 0.34, KO 5.16 ± 0.23, L2-4 WT 6.44 ± 0.3, KO 6.24 ± 0.37; [P14] L1 WT 6.15 ± 0.24, KO 6.48 ± 0.33, L2-4 WT 6.82 ± 0.27, KO 7.22 ± 0.22; [P28] L1 WT 7.05 ± 0.27, KO 6.89 ± 0.25, L2-4 6.75 ± 0.17, KO 7.34 ± 0.41). To analyze cell density in the VC, we quantified cells labeled by nuclear marker DAPI. Cell density decreased across development in accordance with our previous work^4^, and did not differ between WT and KO groups (data shown as mean ± s.e.m. [cell number per mm^2^]: [P7] WT 4504 ± 253.7, KO 4186 ± 120.5, [P14] WT 2274 ± 99.58, KO 2262 ± 101.2, [P28] WT 2275 ± 221.1, KO 2221 ± 230.1; Fig. S3A). To examine how axonal and cell density are affected by IP3R2 KO in regions outside the VC, we quantified NF200 and DAPI density in the Schaffer collaterals of the hippocampus (Fig. S3B-F) and similarly, observed no differences between WT and KO groups (data shown as mean ± s.e.m. NF200 fluorescent signal [thresholded area mm^2^]: WT 13424 ± 887.4, KO 13366 ± 1067 Fig. S3C; skeleton density [number of fragments per mm^2^]: WT 2155 ± 146.5, KO 2322 ± 116.5 Fig. S3D; axonal fragment length [µm]: WT 6.04 ± 0.32, KO 5.52 ± 0.28 Fig. S3E; DAPI [cell number per mm^2^]: WT 2376 ± 187.4, KO 2329 ± 59.76; Fig. S3F). These results suggest that the developmental synaptic protein deficiencies observed in IP3R2 KO mice VC are likely to be synapse-specific, and not due to an overall decrease in cell numbers or gross axonal morphology.

### Inhibitory synapse development in the visual cortex is largely unaffected by IP3R2 knockout

**Fig. 3:**
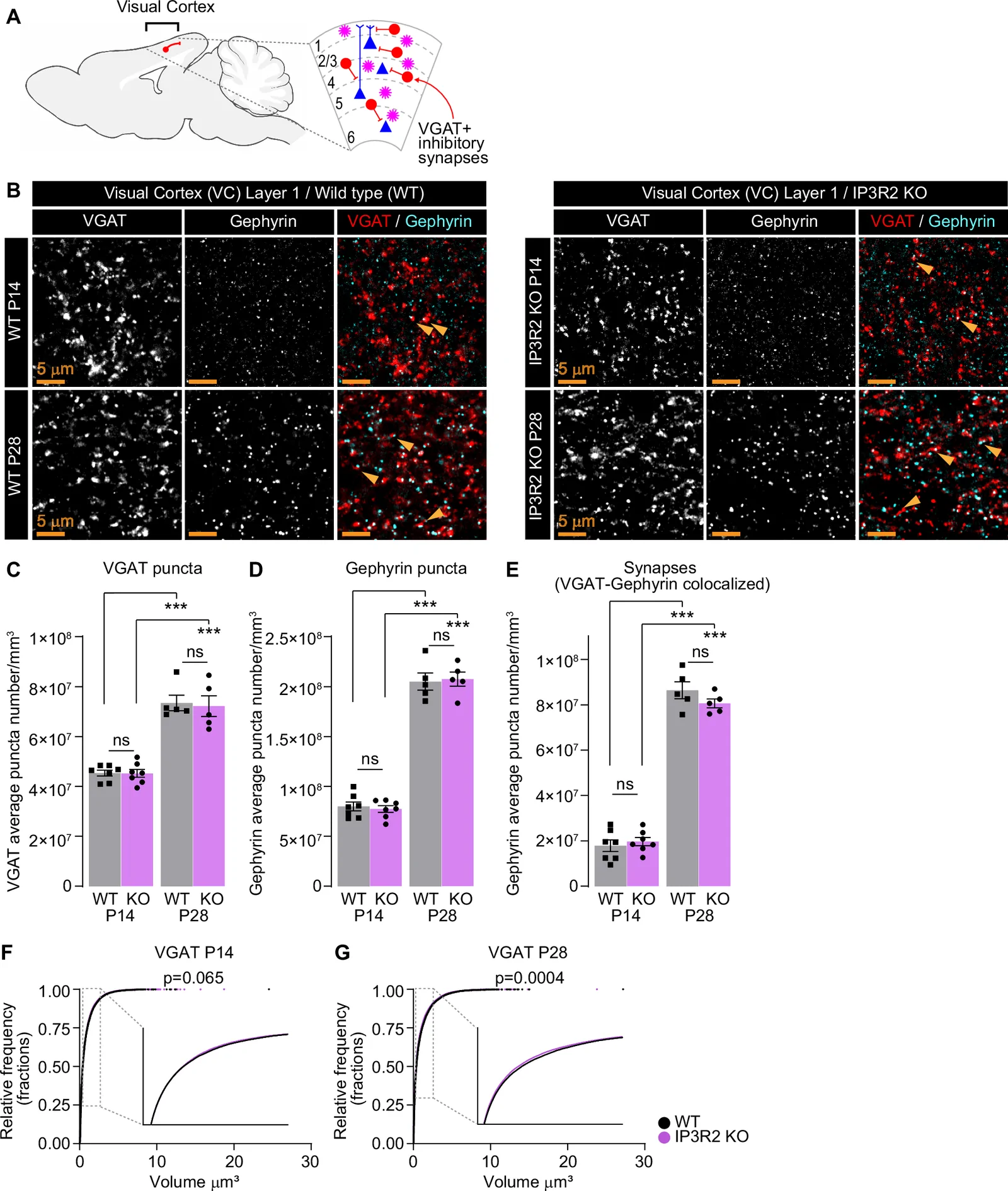
GABAergic synapse numbers are unchanged, VGAT volumes slightly reduced in IP3R2 KO mice VC. See also Supplementary Data 1. **A** Diagram depicting inhibitory neurons within the VC L1 analyzed. **B–E** GABAergic synapse numbers are not altered in IP3R2 KO VC at P14 or P28. **B** Example images of the presynaptic VGAT, postsynaptic Gephyrin and colocalized (synapses) in each genotype as labeled. Single channel grayscale images on the left, merged images on the right. **C–E** Quantification of each synaptic protein (**C**–**D**) and synapse number per mm^3^ represented as colocalization between VGAT and Gephyrin for both genotypes (**E**) are shown. Synaptic proteins as well as synapse numbers are developmentally increased similarly for WT and KO mice, with no difference between genotypes. Graphs show mean ± s.e.m.; individual mouse data points are shown as circles and squares. Statistical analysis by two-way ANOVA with multiple comparison correction by the two-stage linear step-up procedure of Benjamini, Kriger and Yekutieli examining the effects of age and genotype; ***P < 0.001 comparing age groups within each genotype; *P* value (p) on the plots comparing WT and KO groups. ns denotes non-significant results (*P* > 0.05). **F**,** G** Cumulative distributions of volumes from 3D rendered images for VGAT per age and genotype as labeled. Increased fraction of smaller volumes ( < 5 µm^3^) is observed in IP3R2 KO mice at P28. *P* value (p) on the plots comparing WT and KO groups by Kolmogorov-Smirnov test. Number of mice/group (N) *N* = 7 for P14, *N* = 5 for P28. Scale bar = 5 μm. Arrowheads mark representative colocalized puncta. Descriptive statistics and detailed statistical test results for all data panels are provided in Supplementary Data 1.

The mouse VC is composed of ~80% glutamatergic and ~20% gamma-aminobutyric acid (GABA)-ergic neurons^46^. To test whether the observed synaptic deficits are specific to excitatory circuits we used IHC to label presynaptic vesicular GABA transporters (VGAT) and the inhibitory postsynaptic scaffolding protein Gephyrin in WT and IP3R2 KO mice at P14 and P28 (Fig. 3A, B), the timepoints at which we observed deficits in glutamatergic synapses (Fig. 1C–J; Fig. S2A–H). Inhibitory synapse numbers significantly increased with age, consistent with previous studies^47,48^. At either age, there was no difference between WT and KO groups in VGAT puncta number (shown as mean ± s.e.m. [puncta number per mm³]: P14 WT 4.53E7 ± 1.18E6, KO 4.52E7 ± 1.58E6, P28 WT 7.35E + 07 ± 3.13E + 06, KO 7.22E + 07 ± 4.13E + 06; Fig. 3C), Gephyrin puncta number (P14 WT 8.00E7 ± 4.40E6, KO 7.73E7 ± 3.36E6, P28 WT 2.05E + 08 ± 8.64E + 06, KO 2.07E + 08 ± 7.09E + 06; Fig. 3D), or synapse number (VGAT-Gephyrin colocalized) (P14 WT 1.79E7 ± 2.54E6, KO 1.20E7 ± 1.79E6, P28 WT 8.64E + 07 ± 3.75E + 06, KO 8.06E + 07 ± 1.96E + 06; Fig. 3E). However, VGAT volumes were slightly reduced, by ~6% in KO group, statistically significant at P28 (shown as mean ± s.e.m. [volume µm³]: P14 WT 0.80 ± 0.007, KO 0.75 ± 0.006; Fig. 3F, P28 WT 0.93 ± 0.01, KO 0.87 ± 0.009; Fig. 3G). These results demonstrate that developmental disruptions of structural synapses in IP3R2 KO mice are mainly restricted to glutamatergic terminals in the VC, with lesser or potentially later impact on inhibitory synapses.

### Visually evoked upregulation of neuronal immediate early gene c-FOS is blunted in IP3R2 knockout visual circuits

**Fig. 4:**
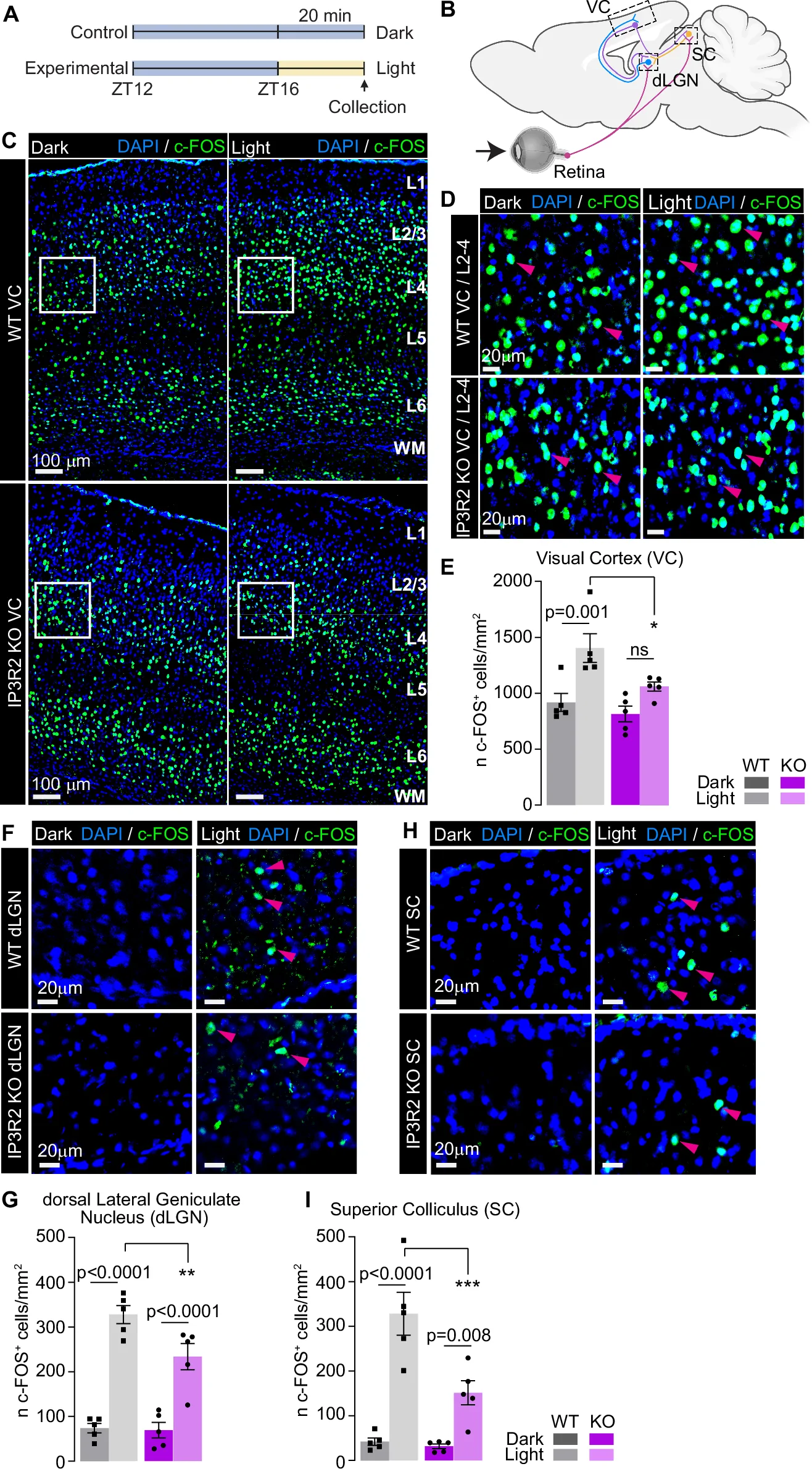
Light-induced c-FOS expression is blunted in IP3R2 KO mice visual circuit. See also Figure S4, Supplementary Data 1. **A** Schematic of the experimental paradigm. Following 4 h of dark exposure (ZT12 marks lights off), mice were exposed to a 20-min light pulse, tissue collected immediately after for c-FOS expression analysis by IHC. **B** Diagram of the visual circuit components including the retina (which receives the light input) and downstream connections to the superior colliculus (SC) and dorsal Lateral Geniculate Nucleus (dLGN) of the thalamus, which input to the Visual cortex (VC). **C–E** Light evoked c-FOS levels are diminished in IP3R2 KO mice VC. **C** Example images of the cortex including all cortical layers as labeled showing c-FOS (green) and nuclear marker DAPI (blue) in each genotype in the dark or light conditions. **D** Zoomed in images from box in (**C**) as labeled showing Layers (L) 2–4. **E** Quantification of (**C**) represented as numbers of c-FOS labeled cells per area in mm^2^ (c-FOS cell density). Light exposure induces a significant increase in c-FOS density in the WT, but not KO VC. **F**–**I** same as (**C**–**E**) but for the dLGN (**F**, **G**) and the SC (**H**, **I**). In both regions, light exposure produced a strong increase in c-FOS positive cell density in the WT, and to a lesser extent in the KO. Graphs show mean ± s.e.m. Individual mouse data points are shown as circles and squares. Number of mice/group (N) *N* = 5. Scale bar in (**B**) = 100 μm; in (**D**), F, H = 20 μm. Arrowheads mark representative c-FOS positive cells. Statistical analysis by two-way ANOVA with multiple comparison correction by the two-stage linear step-up procedure of Benjamini, Kriger and Yekutieli examining the effects of light exposure and genotype; *P ≤ 0.05; **P < 0.01; ***P < 0.001 comparing the effect of light pulse within each genotype; P value (p) on the plots comparing WT and KO groups. ns denotes non-significant results (*P* > 0.05). Descriptive statistics and detailed statistical test results for all data panels are provided in Supplementary Data 1.

Reduction in excitatory synaptic proteins in the VC could contribute to deficits in visually evoked neuronal activity, which has been shown to undergo regulation by astrocyte Ca^2+^ fluctuations^4,40^. To test whether disruption of IP3R2-mediated store-released Ca^2+^ affects the activation of neurons in the VC, we examined activity-dependent expression of the protein product of the immediate early gene *c-fos* (protein name c-FOS) in response to a brief light pulse stimulus following prolonged darkness (4 h; Fig. 4A). Fluorescent immunolabeling of c-FOS protein in the VC revealed that in WT mice, light stimulation induced a robust increase in the number of c-FOS positive cells ( ~ 50%) compared to control group kept in the dark (shown as mean ± s.e.m. [cell number per mm^2^]: WT dark 917.4 ± 80.38, light 1404 ± 127.9; Fig. 4C–E). Because distinct cortical layers receive and integrate different streams of visual input and output (Figs. 1A, 4B, and^4^), we also analyzed c-FOS induction separately across layers (Fig. S4A–E). We observed increased expression across the cortical gray matter, with statistically significant changes in L2/3 ( ~ 80%) which integrates local input response from other layers, L4 ( ~ 40%) which receives visual input from the thalamus, as well as the output layers (L5 ~ 50% and L6 ~ 30%) (WT [L1] dark 41.81 ± 5.92, light 80.90 ± 27.21, [L2/3] dark 892.7 ± 63.37, light 1649 ± 107, [L4] dark 1512 ± 143.6, light 2090 ± 170.9, [L5] dark 770.5 ± 81.37, light 1180 ± 164, [L6] dark 1247 ± 131.5, light 1654 ± 168.9; Fig. S4A–E).

In the IP3R2 KO VC this effect was blunted, showing weaker light-induced c-FOS expression ( ~ 20–30% increase compared to dark condition; statistically significant in L1 and L2/3), which was significantly reduced compared to WT in both total VC and Layers 2/3 and 4 (KO [VC] dark 814.2 ± 69.86, light 1059 ± 41.08; Fig. 4C–E; [L1] dark 31.59 ± 7.42, light 82.92 ± 16.74, [L2/3] dark 764.1 ± 112.1, light 1135 ± 98.36, [L4] dark1424 ± 77.12, light 1676 ± 79.47, [L5] dark 657.8 ± 92.57, light 924.5 ± 78.37, [L6] dark 1088 ± 96.1, light 1313 ± 69.65; Fig. S4A–E). Notably, no difference in c-FOS expression was observed between WT and IP3R2 KO in the control (dark) condition, suggesting that basal level of neuronal activation is not perturbed by IP3R2 removal. The observed effects were not due to changes in cell number as evident from similar DAPI-positive signal across the genotypes and light/dark conditions (data shown as mean ± s.e.m. [cell number per mm^2^]: [L1] WT dark 1360 ± 42.84, light 1430 ± 83.73; KO dark 1420 ± 70.53, light 1281 ± 63.49; [L2/3] WT dark 2879 ± 113.5, light 3069 ± 212.2; KO dark 2911 ± 86.31, light 2790 ± 201.8; [L4] WT dark 3377 ± 324, light 3801 ± 283.6; KO dark 3162 ± 190.8, light 2916 ± 252.3; [L5] WT dark: 2523 ± 202.8, light 2201 ± 182.4; KO dark 2678 ± 220.4, light 2414 ± 348.7 [L6] WT dark 3724 ± 281, light 3617 ± 225.4; KO dark 3444 ± 343.9, light 3207 ± 279.1; Fig. S4J–O).

It has been shown that c-FOS can be also expressed by non-neuronal cells (specifically astrocytes)^49–51^. To test whether the observed deficits were specific to neuronal activation, we quantified cell type specific expression of c-FOS in the VC by co-labeling WT sections with Neun and S100β to mark neurons and astrocytes respectively and analyzed the data both by manual count of regions of interest (ROIs; Fig. S4G) and semi-automated thresholding analysis of larger cortical area (Fig. S4H). We observed robust co-expression of c-FOS with the neuronal marker Neun, and minimal co-labeling with S100β in both dark and light conditions with ~95% of c-FOS+ cells co-expressing Neun, while the remaining ~5% did not colocalize with either neuronal or astrocytic marker by manual count analysis (Fig. S4I) (data shown as mean ± s.e.m. [cell number per mm^2^]: manual count c-FOS+Neun+ dark 9.66 ± 2.51, light 40.78 ± 0.96, c-FOS + S100β+ dark 0 ± 0, light 0 ± 0; threshold analysis: c-FOS+Neun+ dark 1042 ± 272.3, light 2784 ± 68.71, c-FOS + S100β+ dark 30.06 ± 19.1, light 133 ± 36.05; Fig. S4G-H). These results demonstrate that in the developing VC at P16, c-FOS expression is almost exclusively restricted to neurons.

To explore whether attenuated activity in the KO VC reflects upstream changes in subcortical visual processing, we examined c-FOS expression in the dorsal lateral geniculate nucleus (dLGN) (Fig. 4F, G) and superior colliculus (SC) (Fig. 4H, I) which receive direct retinal input and relay visual information to the VC^52–55^ (Fig. 4B). WT mice exhibited dramatic increases in c-FOS positive cell numbers in both the dLGN ( ~ 340%) and SC ( ~ 680%) upon light stimulation (WT [dLGN] dark 73.99 ± 10.67, light 328.9 ± 20.64, [SC] dark 41.93 ± 8.107, light 328.2 ± 48.14; Fig. 4G, I). However, this increase was blunted in IP3R2 KO group showing ~240% increase in the dLGN and ~380% in the SC, significantly lower than in WT mice in both regions (KO [dLGN] dark 69.13 ± 17.36, light 233.8 ± 29.52 [SC] dark 31.80 ± 5.31, light 151.5 ± 26.94; Fig. 4G, I). Like in the VC, these c-FOS changes were not due to differences in cell numbers between WT and IP3R2 KO mice as evident from similar DAPI signal (WT [dLGN] dark 3074 ± 61.99, light 3091 ± 197.4; [SC] dark 3427 ± 196.8, light 3463 ± 196.1; KO [dLGN] dark 2487 ± 159.8, light 2871 ± 285.5; [SC] dark 3240 ± 134.6, light 3400 ± 146.6; Fig. S4P–Q). To validate that the effects of the light pulse were vision-specific, we quantified the number of c-FOS positive cells in the CA1 and dentate gyrus (DG) regions of the hippocampus (Fig. S4T–V) and observed no changes in c-FOS protein induction, or DAPI-labeled cell numbers (Fig. S4R-S) between the genotypes or light/dark conditions, as expected (c-FOS signal WT [CA1] dark 1532 ± 233.9, light 1992 ± 365.5; [DG] dark 309.6 ± 32.1, light 369.4 ± 37.41; KO [CA1] dark 1324 ± 254, light 1642 ± 145; [DG] dark 295.0 ± 44.34, light 288.1 ± 40.27; Fig. S4U–V; DAPI signal WT [CA1] dark 5812 ± 439.8, light 5194 ± 473.5; [DG] dark 4416 ± 200.4, light 4938 ± 312.3; KO [CA1] dark 4683 ± 527.3, light 4418 ± 510.3; [DG] dark 4416 ± 152.5, light 4241 ± 375.8; Fig. S4R-S). These results indicate that IP3R2-mediated Ca^2+^ signaling plays an important role in facilitating visually evoked, but not basal, neuronal activity across brain regions comprising the visual circuit.

### Visually evoked defensive behavioral responses are disrupted in IP3R2 knockout mice

**Fig. 5:**
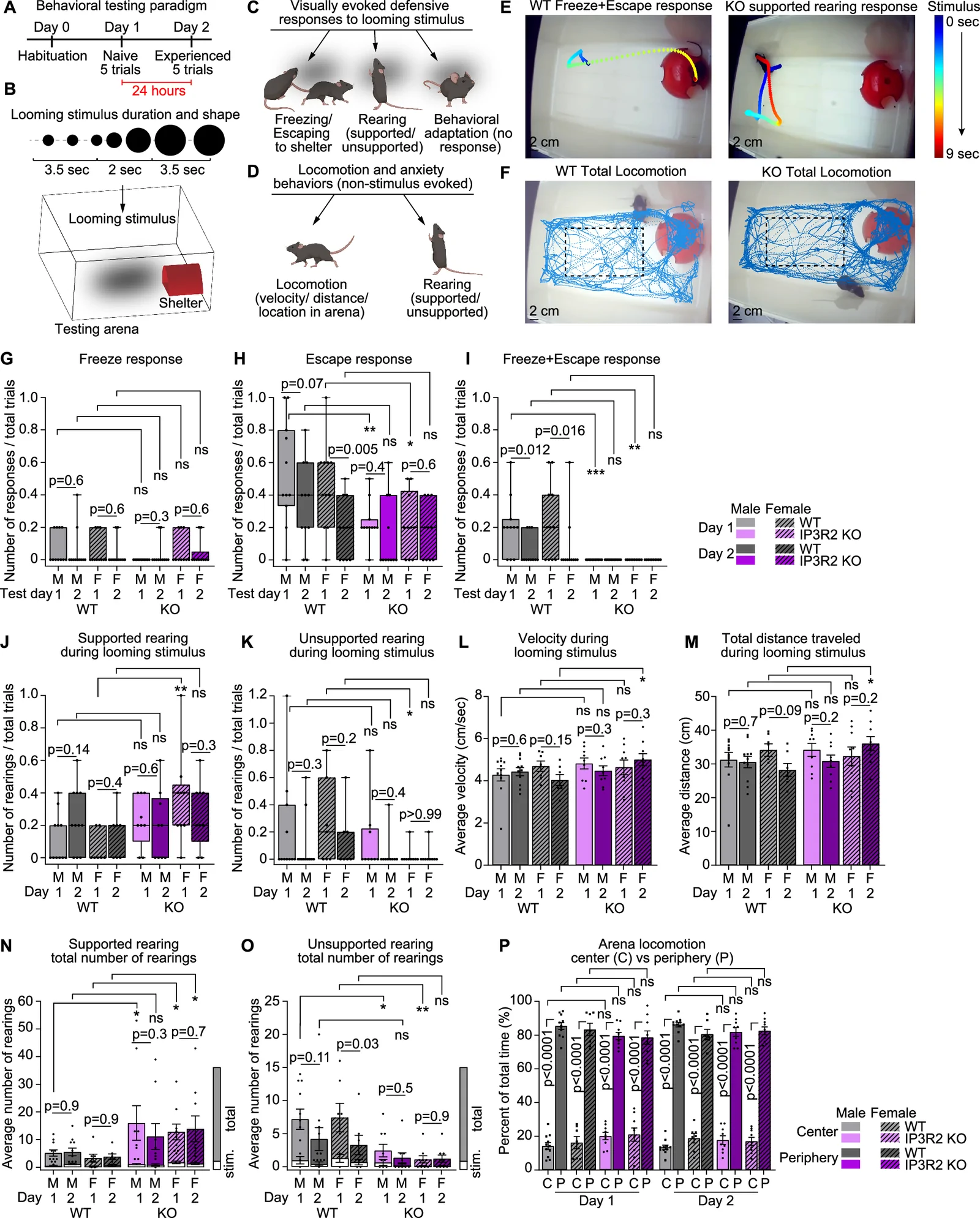
Visually evoked defensive responses and rearing behaviors are disrupted in IP3R2 KO mice. See also Fig. S5, Supplementary Data 1. **A** Diagram of the behavioral experiment including habituation and 2 testing days performed 24 h apart. Each testing day includes 5 trials of the looming stimulus. Mice are naïve to the stimulus on Day 1 and retested on Day 2 (experienced). **B** Schematic of the looming stimulus shape and duration (top) and testing arena showing location of the escape shelter (red hut) and the perceived “shadow” created by the stimulus (bottom). Diagrams of the types of defensive behaviors in response to the looming stimulus (**C**) and general locomotion and anxiety-related behaviors (**D**) analyzed. **E** Example frame images from recorded videos showing mouse behavioral responses in WT (left, “Freeze+Escape” response) and KO (right, “Supported rearing” behavior) occurring during the looming stimulus. Frame by frame DeepLabCut tracking is shown colorcoded by stimulus time progression (scale bar is on the right). **F** Example of DeepLabCut tracing used to analyze mouse general locomotion and arena localization (open field center vs periphery analysis) showing no difference in overall ambulatory abilities between WT (left) and KO (right) mice. Scale bar = 2 cm. **G–K** Average responses recorded during the looming stimulus as labeled across genotypes, sexes, and experimental days. “Freeze” response is similar between testing days, sex and genotypes (**G**). WT mice “Escape” responses are reduced on Day 2 compared to Day 1 and are significantly lower in KO group compared to WT for both sexes on Day 1 (**H**). “Freeze+Escape” responses are reduced in WT mice on Day 2 but are absent in KO mice (**I**). “Supported rearing” during stimulus is higher in KO mice (significant in females) compared to WT with no difference between testing days (**J**). “Unsupported rearing” during stimulus is reduced in KO compared to WT (significant in females), with no difference between testing days (**K**). Velocity (**L**) and total distance traveled (**M**) averaged across the looming stimulus, shows similar locomotor activity between testing days for each sex, with a significantly increased velocity and distance in KO females compared to WT females on Day 2. Analysis of rearing events (supported (**N**) and unsupported (**O**)) during the total testing period showing significantly increased “Supported rearing” and significantly lower “Unsupported rearing” in KO mice compared to WT. White bars within each graph depict the number of rearing events occurring during the looming stimulus (as related to total number of rearing events) for each genotype, sex and testing day as labeled. **P** Analysis of time spent in center vs periphery of the arena showing significantly higher localization preference for both sexes and genotypes to periphery over the center. In (**G**–**K**), graphs show box with range, line is median. Individual mouse data points are shown as circles. In (**L**–**P**), graphs show mean ± s.e.m; individual mouse data points are shown as circles or squares. Number of mice (N) *N* = 7–11/sex/genotype. Statistical analysis by two-way ANOVA with multiple comparison correction by the two-stage linear step-up procedure of Benjamini, Kriger and Yekutieli examining the effects of testing day and genotype; *P ≤ 0.05; **P < 0.01; ***P < 0.001 comparing between WT and KO groups within each sex; P value (p) on the plots comparing Day 1 and Day 2 responses within each sex and genotype. ns denotes non-significant results (*P* > 0.05). Descriptive statistics and detailed statistical test results for all data panels are provided in Supplementary Data 1.

Since we observed circuit level deficits in visually evoked neuronal activation, we next asked whether these findings translate to alterations in the development of behaviors which rely on these circuits for appropriate execution. Visually evoked defensive responses depend on an animal’s ability to perceive the threatening stimulus and integrate that information to coordinate an appropriate behavioral output^56,57^. We hypothesized that if IP3R2 KO mice showed significantly reduced levels of excitatory synapse markers (Fig. 1G, J) and experienced reduced or delayed activation of visual circuit neurons in response to salient stimuli (Fig. 4), they may exhibit disrupted behavioral responses which require intact signaling in the visual circuitry. To test this hypothesis, we subjected 4-week-old (P30) WT and IP3R2 KO mice to a visual looming threat paradigm (at this age, WT mice robustly respond to the looming threat according to our previous work^58^, while synaptic deficits persist in the IP3R2 KO; Fig. 1). During this task, mice are presented with a visual stimulus in a form of expanding black circle on a white background projected from above, which mimics a looming predator^59–61^ (Fig. 5B, see “Methods” and^58^), evoking distinct defensive responses including: “Freeze”—mice exhibit immobility; “Escape”—mice move towards and enter the shelter; “Freeze+Escape”—immobility followed by movement towards and entering the shelter (Fig. 5E, WT). Additional behaviors including “Supported rearing” – mice use the arena walls or other objects to stand on hind paws (Fig. 5E, KO), “Unsupported rearing” – mice stand on hind paws without using arena walls or objects for support (Fig. 5C, D), and locomotion (e.g., velocity, distance traveled, arena localization; Fig. 5F) both during and outside of the stimulus were also quantified to assess general ambulatory abilities, states of alertness, anxiety and exploratory drive (which can impact defensive responses). The test was administered over 3 consecutive days and included a habituation day (no stimulus) and 2 testing days (5 stimuli per day), where on Day 1 mice are naïve to the stimulus, while 24 h later (Day 2), mice are retested to examine behavioral adaptation (Fig. 5A). Defensive and rearing behaviors in response to the looming stimulus were scored as 1—response, 0—no response (mice continue exploratory behavior such as movement or sniffing) and averaged for each individual (see “Methods”).

Wild type mice of both sexes (M/F) robustly responded to the looming stimulus exhibiting various response types including “Escape” (Fig. 5H), “Freeze+Escape” (Fig. 5I), “Unsupported rearing” (Fig. 5K), “Freeze” (Fig. 5G), and “Supported rearing” (Fig. 5J). Responses were typically reduced on Day 2 of testing, indicating behavioral adaptation (statistically significant for “Escape” and “Freeze+Escape” responses; Fig. 5H, I). In IP3R2 KO mice, “Escape” and “Unsupported rearing” responses were significantly lower compared to WT ( ~ 50% reduction in average “Escape” response in both sexes (Fig. 5H), and ~50% reduction in “Unsupported rearing” in both sexes, statistically significant in Females (F); Fig. 5K), while “Freeze+Escape” responses were undetected in KO mice of either sex (Fig. 5I). Conversely, “Freeze” responses did not differ between genotypes (Fig. 5G), while “Supported rearing” responses were higher in the KO group (M ~ 88%, F ~ 630%, statistically significant in Females (F); Fig. 5J). Moreover, no difference between Day 1 (D1) and Day 2 (D2) responses were observed in the KO groups for any response type, suggesting lack of behavioral adaptation (Average responses data shown as median and [range]: “Freeze” WT M D1 0 [0–0.2], D2 0 [0–0.4]; F D1 0 [0–0.2], D2 0 [0–0.2] KO M D1 0 [0–0], D2 0 [0–0.2]; F D1 0 [0–0.2], D2 0 [0–0.2]; Fig. 5G; “Escape” WT M D1 0.4 [0–1.0], D2 0.4 [0–0.8]; F D1 0.4 [0–1.0], D2 0.2 [0–0.5]; KO M D1 0.2 [0–0.5], D2 0 [0–0.6]; F D1 0.2 [0–0.5], D2 0.2 [0–0.4]; Fig. 5H; “Freeze+Escape” WT M D1 0.2 [0.0–0.6], D2 0 [0–0.2]; F D1 0.2 [0–0.6], D2 0 [0–0.6]; KO M D1 0 [0–0], D2 0 [0–0]; F D1 0 [0–0], D2 0 [0–0]; Fig. 5I; “Supported rearing” WT M D1 0 [0–0.4], D2 0.2 [0–0.6]; F D1 0 [0–0.2], D2 0.2 [0–0.4]; KO M D1 0.2 [0–0.4], D2 0.2 [0–0.6]; F D1 0.4 [0–1], D2 [0–0.6]; Fig. 5J; “Unsupported rearing” WT M D1 0 [0–1.2], D2 0 [0–0.6]; F D1 0.2 [0–0.8], D2 0 [0–0.6]; KO M D1 0 [0–0.8], D2 0 [0–0.4]; F D1 0 [0–0.2], D2 0 [0–0.2]; Fig. 5K).

Overall, the “Escape” response was most frequent in WT mice, while “Supported rearing” was most frequent response in KO suggesting distinct defensive strategies between the genotypes (Percentage of different responses out of total response number per day: “Freeze” WT M D1 7.69%, D2 5.45%; F D1 5.55%, D2 3.77%; KO M D1 0%, D2 3.92%; F D1 6.25%, D2 4%; “Escape” WT M D1 53.84%, D2 34.54%; F D1 44.44%, D2 18.86%; KO M D1 22.64%, D2 15.68%; F D1 20.83%, D2 18%; “Freeze+Escape” WT M D1 21.15%, D2 5.45%; F D1 24.07%, D2 7.54%; KO M D1 0%, D2 0%; F D1 0%, D2 0%; “Supported rearing” WT M D1 11.53%, D2 25%; F D1 3.84%, D2 9.61%; KO M D1 19.23%, D2 15.38%; F D1 30.76%, D2 23.07%; “Unsupported rearing” WT M D1 21.15%, D2 9.61%; F D1 17.3%, D2 9.61%; KO M D1 11.53%, D2 3.84%; F D1 1.92%, D2 1.92%; Fig. S5J).

To determine responsiveness at the group level, we quantified the percent responding mice (represented as mice who respond to at least one trial per day) out of total group number and observed no difference between WT and IP3R2 KO groups of either sex across test days for “Freeze” response (WT M D1 36%, D2 18%, F D1 27%, D2 18%; KO M D1 0%, D2 18%, F D1 30%, D2 20%; Fig. S5A), “Escape” response (WT M D1 91%, D2 82%, F D1 91%, D2 64%; KO M D1 91%, D2 36%, F D1 70%, D2 60%; Fig. S5B), or “Unsupported rearing” response (WT M D1 36.36%, D2 18%; F D1 57.14%, D2 42.85%; KO M D1 33.33%, D2 11.11%; F D1 11.11%, D2 11.11%; Fig. S5E). Conversely, percent responders by “Freeze+Escape” is significantly higher in WT group, with none of the KO mice exhibiting this response type, in accordance with our average response data (WT M D1 73%, D2 27%, F D1 64%, D2 64%; KO M D1 0%, D2 0%, F D1 0% D2 0%; Fig. S5C), while “Supported rearing” was detected in higher percentage of KO mice than WT (statistically significant in naïve females; WT M D1 45.45%, D2 72.72%; F D1 28.57%, D2 57.14; KO M D1 77.77%, D2 55.55%; F D1 88.88%, D2 77.77%; Fig. S5D). These results suggest that the altered responses in KO mice are not stemming from abnormal response from select individuals, but rather, a group-wide changes in responsiveness.

Importantly, average velocity and distance traveled during the stimulus (Fig. 5L, M) as well as during the full testing period (Fig. S5H–I) were similar overall across the experimental groups (slightly increased in KO females), suggesting that the observed responses were not due to differences in ambulatory abilities (shown as mean ± s.e.m velocity during stimulus [cm/sec]: WT M D1 4.28 ± 0.28, D2 4.43 ± 0.19, F D1 4.69 ± 0.24, D2 4.03 ± 0.25; KO M D1 4.82 ± 0.26, D2 4.47 ± 0.23, F D1 4.64 ± 0.35, D2 5.004 ± 0.28; Fig. 5L; velocity total: WT M D1 3.17 ± 0.13, D2 3.18 ± 0.2; F D1 3.14 ± 0.32, D2 3.09 ± 0.26; KO M D1 3.54 ± 0.26, D2 3.34 ± 0.36; F D1 3.73 ± 0.34, D2 3.92 ± 0.34; S5H; distance traveled during stimulus [cm]: WT M D1 31.24 ± 2.14, D2 30.56 ± 1.69; F D1 34.12 ± 1.86, D2 28.22 ± 1.92; KO M D1 34.18 ± 1.91, D2 30.83 ± 1.8; F D1 32.32 ± 2.71, D2 36.02 ± 2.08; Fig. 5M; distance total: WT M D1 1744 ± 101.1, D2 1623 ± 125; F D1 1624 ± 127.3, D2 1670 ± 101.8; KO M D1 1979 ± 187.3, D2 1928 ± 244; F D1 2226 ± 225.5, D2 2320 ± 237; Fig. S5I). To analyze mouse anxiety state, we calculated the percentage of time mice spent in the center versus periphery of the arena (Fig. 5F, P) and observed that all mice spent more time in the periphery (Fig. 5P; (P), ~80%) than in the center ((C) ~ 20%) with no differences between genotypes, sex, or testing days (Fig. 5P). We further quantified the average number of supported and unsupported rearing events throughout the total testing period (up to 13 min) and observed significantly higher numbers of “Supported rearing” (shown as mean ± s.e.m: WT M D1 5.18 ± 1.11, D2 5.54 ± 1.39; F D1 3.28 ± 1.42, D2 3.85 ± 0.98, KO M D1 16 ± 6.26, D2 11.11 ± 4.67; F D1 12.78 ± 2.83, D2 13.89 ± 4.66; Fig. 5N), and significantly lower numbers of “Unsupported rearing” in the KO groups, consistent with stimulus-related data in Fig. 5J–K (shown as mean ± s.e.m: WT M D1 7.18 ± 1.53, D2 4.18 ± 1.73; F D1 7.42 ± 2.15, D2 3.28 ± 1.47; KO M D1 2.44 ± 0.95, D2 1.33 ± 0.76; F D1 1.11 ± 0.53, D2 1.22 ± 0.54; Fig. 5O). Interestingly, while virtually all mice exhibited the “Supported rearing” behavior during the testing period, albeit at different magnitudes ( ~ 75–100% of mice reared at least once; Fig. S5F), the percentage of mice exhibiting “Unsupported rearing” was lower in KO mice (by ~30%), statistically significant for D2 males; Fig. S5G, signifying reduced responsiveness at the individual level as well as the group.

These findings demonstrate that IP3R2 KO mice exhibit impairment in visually evoked defensive behaviors, despite normal mobility and anxiety levels during aversive visual stimuli, supporting a role for astrocytic store-released Ca^2+^ signaling in shaping behavior through experience-dependent circuit refinement.

### Astrocyte morphogenesis and synapse contact is perturbed in IP3R2 knockout visual cortex

**Fig. 6:**
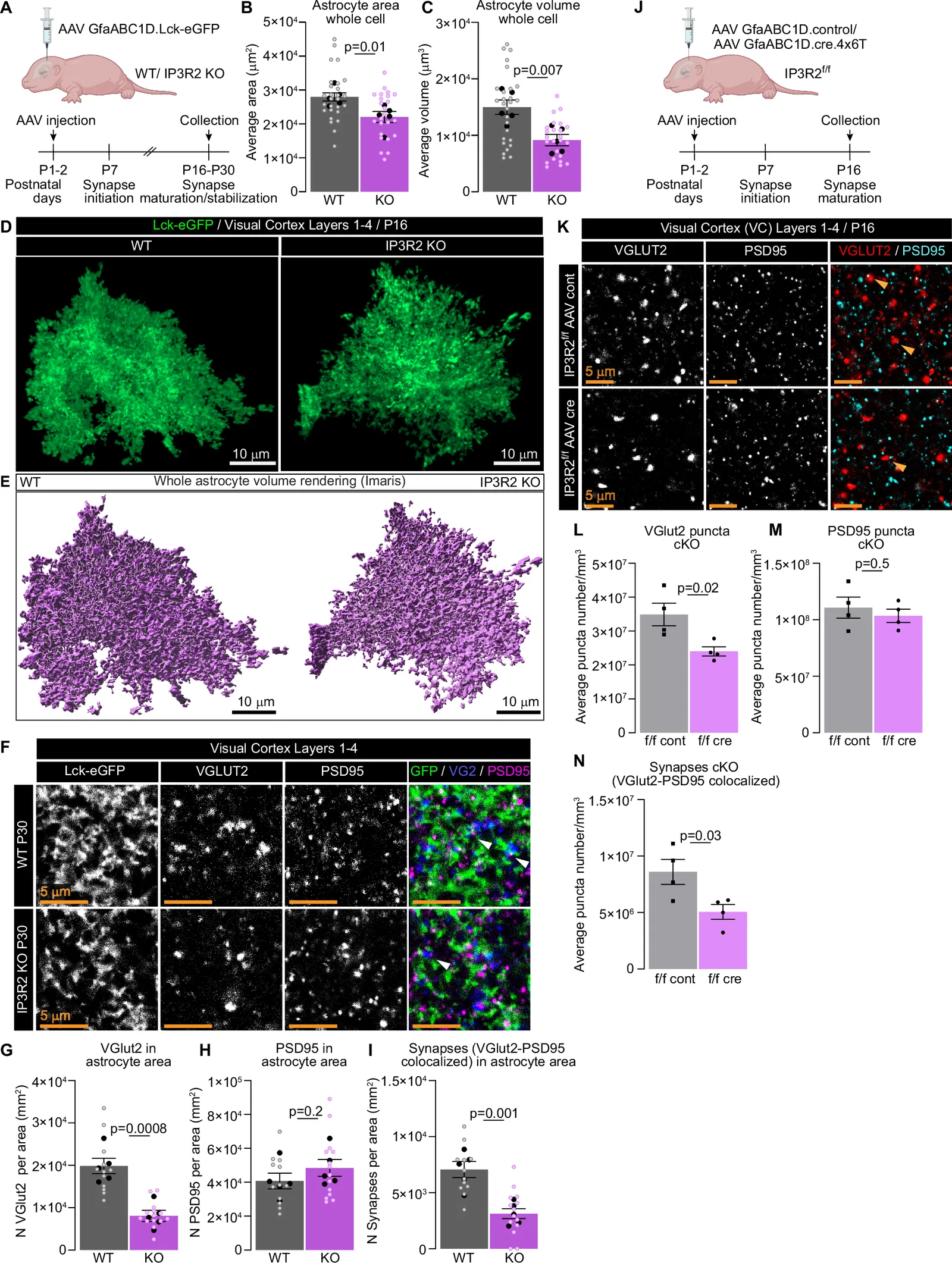
Reduced astrocytic morphogenesis and synapse contact in germline IP3R2 KO mice, and reduced synapse numbers in astrocyte-specific conditional IP3R2 cKO VC. See also Fig. S6, Supplementary Data 1. **A** Schematic of the strategy to express fluorescent reporter in astrocytes. P1-2 mouse pups injected intracerebroventricularly (ICV) with AAV to express membrane tethered eGFP (Lck-eGFP) under astrocyte specific promoter GfaABC1D. Tissue collected 2 or 4 weeks following injection was sectioned and imaged using super resolution confocal microscope (Zeiss Airyscan; see “Methods”). **B–E** Whole cell astrocytic volume and area are reduced in IP3R2 KO mice compared to WT at P16. Example images of Lck-eGFP (green) expressing astrocytes (**D**) and 3D rendering of volume using Imaris (**E**) for each genotype is shown as labeled. **B**,** C** Quantification shows reduced total volume and area of IP3R2 KO astrocytes in the VC. **F–I** Astrocyte volume and synapse numbers within astrocyte territories are reduced in IP3R2 KO astrocytes at P30. **F** Example zoomed in images from box (Fig. S6H) of Lck-eGFP injected astrocytes (GFP, green) co-labeled with antibodies for synaptic markers VGLUT2 (VG2, blue) and PSD95 (magenta) in each genotype as labeled. **G**,** H** Quantification of (**F**), showing reduced number of VGLUT2 puncta (**G**), No change in PSD95 puncta (**H**), and reduced number of synapses (VGLUT2-PSD95 colocalized) within the territory of KO astrocytes (**I**). **J** Schematic of the strategy to postnatally knockout IP3R2 from astrocytes via AAV delivery through ICV injection (as in (**A**)), to express cre recombinase under the astrocyte specific promoter GfaABC1D into P1-2 IP3R2 fl/fl mice (see “Methods”). The AAV plasmid includes the inhibitory 4x6T cassette to reduce off-target viral expression. **K–N** Glutamatergic synapse number is reduced in IP3R2 conditional knockout (cKO) mice VC at P16. Example images of the presynaptic VGLUT2, postsynaptic PSD95 and merged (synapses) in each genotype as labeled (**K**), and quantification of individual synaptic proteins and synapse number per mm^3^ represented as colocalization between VGLUT2 and PSD95 for both genotypes (**L**–**N**) are shown. Single channel grayscale images on the left, merged images on the right. The number of VGLUT2 puncta and VGLUT2-PSD95 colocalizations is reduced in IP3R2 cKO (**L**, **N**); PSD95 numbers are unaltered (**M**). Graphs show mean ± s.e.m. Black circles or squares are average of signal in each individual mouse; colored open circles in (**B**, **C**, **G**–**I**) are data for each astrocyte. Number of mice/group (N) in (**B**, **C**, **G**–**I**) *N* = 5; number of astrocytes = 25–27, in (**L**–**N**) number of mice *N* = 4. Scale bar in (**D**, **E**) = 10 μm, in (**F**), K = 5 μm. *P* value (p) indicated on each plot, by *t* test. Descriptive statistics and detailed statistical test results for all data panels are provided in Supplementary Data 1.

An important process in development is the establishment of astrocytic morphological complexity, which is essential for effective synaptic ensheathment and support^1,62^. Therefore, it is plausible that the observed neuronal deficits in IP3R2 KO arise from astrocytes’ inability to fully and effectively interact with the developing synapses. Indeed, our previous work identified multiple differentially expressed genes in P14 IP3R2 KO mice VC known in the literature to be related to cellular metabolism and growth^4^ (Fig. S6A). We selected several genes for validation using qPCR in VC tissue from WT and IP3R2 KO mice and confirmed significant expression changes in a number of the candidates, further supporting our hypothesis (shown as mean ± s.e.m [Ct relative to *Gapdh*, normalized to WT]: *Glud1* 1.67 ± 0.23; *Grin2b* 1.63 ± 0.23; *Kcnk2* 1.78 ± 0.26; *Mapk10* 1.59 ± 0.15; Fig. S6B). To test for morphological disruptions in IP3R2 KO mice, we delivered an adeno associated viral (AAV) vector via intracerebroventricular (ICV) injection to sparsely express the membrane tethered fluorescent reporter (Lck-eGFP) under control of the minimal astrocytic promoter GfaABC_1_D^63^ at P1-2 and quantified astrocyte morphological development two or four weeks later at P16 and P30 (Fig. 6A). AAV-transduced sections were also co-labeled with synaptic markers VGLUT2 and PSD95 to quantify synapse numbers within astrocyte territories at P30 to examine synaptic support in the absence of IP3R2 (Fig. S6H). The membrane tethered reporter is necessary to fully capture astrocyte finer morphological complexity as well as synapse proximity though somatic and large branch labeling is obscured, while sparse labeling ensures the ability to analyze individual astrocytes. Lck-eGFP labeling was highly specific (100% of Lck-eGFP expressing cells in VC colocalized with S100β astrocytic marker) and relatively sparse ( ~ 30% of S100β positive (^+^) cells expressed Lck-eGFP) validating our approach for these experiments (Fig. S6C-D). To ensure whole cell morphology was captured (and to avoid biasing our analysis by focusing on select segments of the astrocyte), we imaged cortical sections encompassing the entire volume of the astrocyte ( ~ 40 µm z-stack) using super resolution confocal microscopy followed by 3D volumetric rendering constructed from the Lck-eGFP signal using Imaris (Fig. 6D, E; see “Methods”) and subsequent analysis for size and geometric qualities. We observed a significant reduction in total astrocyte volume ( ~ 40%) in IP3R2 KO astrocytes compared to WT (data shown as mean ± s.e.m [µm^3^]: WT 14980 ± 1270, KO 9148 ± 1011; Fig.6B) and in area ( ~ 20%) (shown as mean ± s.e.m [µm^2^]: WT 28004 ± 1187, KO 22093 ± 1624; Fig. 6C). There were no significant differences in oblate ellipticity (WT 0.51 ± 0.02, KO 0.54 ± 0.03; Fig. S6E), prolate ellipticity (WT 0.30 ± 0.04, KO 0.28 ± 0.04; Fig. S6F), or sphericity (WT 0.11 ± 0.007, KO 0.10 ± 0.005; Fig. S6G) between WT and IP3R2 KO astrocytes.

Next, we asked whether the observed morphological deficits at P16 persist at later developmental stages when synaptogenesis is stabilized, and whether they are accompanied by perturbed synaptic coverage by astrocytes in IP3R2 KO mice. We labeled Lck-eGFP expressing sections for the presynaptic marker VGLUT2 and postsynaptic PSD95 and used super-resolution microscopy and volumetric reconstruction analysis to quantify astrocytic morphological complexity as well as synapse number within astrocyte territories in both WT and KO VCs. A 3 µm z-stack was imaged using Lck-eGFP signal as indicator for stack range centered around the largest portion of the astrocyte territory (Fig. S6H). We observed significantly reduced astrocytic volume ( ~ 40%) in the IP3R2 KO mice sections (data shown as mean ± s.e.m [µm³]: WT 4040 ± 251.5, KO 2691 ± 256.6; Fig.S6J), with no significant change in area (data shown as mean ± s.e.m [µm³]: WT 10012 ± 749.6, KO 8938 ± 411.9; Fig.S6I). Structural synaptic quantification within astrocyte territories (Lck-eGFP signal) revealed significant reduction in VGLUT2 puncta numbers ( ~ 60%; shown as mean ± s.e.m [puncta number per astrocyte area µm^2^]: WT 1.98E4 ± 1.81E3, KO 8.07E3 ± 1.32E3; Fig. 6G), VGLUT2 puncta volume ( ~ 25%; data shown as mean ± s.e.m. [volume µm³]: WT 0.92 ± 0.02, KO 0.7 ± 0.02; Fig. S6K), and VGLUT2-PSD95 colocalized puncta (synapses) ( ~ 60%; shown as mean ± s.e.m [puncta number per astrocyte area µm^2^]: WT 7.07E3 ± 7.18E2, KO 3.14E3 ± 4.45E2; Fig. 6I) with no change in PSD95 puncta number (WT 4.08E4 ± 4.6E3, KO 4.85E4 ± 4.96E3; Fig. 6H), in agreement with our previous data (Fig. 1). These findings demonstrate that IP3R2 KO astrocytes have morphological deficits compared to WT at critical developmental timepoints for astrocytic regulation of synapse development and stabilization, suggesting a potential inability to properly contact synapses which may result in the observed reduction in excitatory synapse markers in the developing VC. Notably, while overall astrocyte size was significantly reduced, the general geometry of IP3R2 KO astrocytes remained consistent with WT astrocytes at P16, suggesting that store-released astrocytic Ca^2+^ signaling modulates astrocyte outgrowth and elaboration while fundamental cytoskeletal and membrane patterning mechanisms are likely preserved.

We further utilized an AAV-mediated approach to postnatally knockout IP3R2 specifically from astrocytes by expressing cre recombinase^64^ (or non-translated control) under the minimal astrocytic promoter GfaABC_1_D^63^ in IP3R2^fl/fl^ mice^65^ at P1-2 via ICV injection (Fig. 6J) to examine the impact on expression levels of excitatory synapse markers VGLUT2 and PSD95 and their colocalization two weeks later at P16 (Fig. 5K-N). To validate the efficiency of this knockout approach, we injected the cre expressing AAV ICV into mice expressing cre-dependent fluorescent reporter tdTomato (B6.Cg-Gt(ROSA)26Sortm14(CAG-tdTomato)Hze/J) and observed ~35% recombination efficiency (as indicated by tdTomato expression) and high specificity of the AAV to astrocytes, showing 100% of tdTomato positive cells co-expressing the astrocytic marker S100β in the VC (Fig. S6L–M). IP3R2 removal was validated by IHC (as in Fig. 1) showing significant reduction of both total ( ~ 50%) and astrocytic (colocalized with S100β, ~70%) signal in AAV-cre injected mice (cKO) compared to control (fl/fl) (in Fig. S6O data shown as shown as mean ± s.e.m. [thresholded area µm^2^]: fl/fl 3.63 ± 0.45, cKO 1.36 ± 0.3; in Fig. S6P data shown as mean ± s.e.m. [thresholded area of signal within ROI 101.4 µm^2^]: fl/fl 101.8 ± 13.48, cKO 48.88 ± 2.52). To quantify the effect of postnatal astrocytic IP3R2 removal on synapse development, we conducted IHC experiments labeling for presynaptic VGLUT2 and postsynaptic PSD95 (Fig. 6F) and, as in the germline KO mice (Fig. 1), we observed significantly reduced VGLUT2 puncta numbers ( ~ 30%; Fig. 6K, L) and volume ( ~ 12%; Fig. S6Q) in AAV-cre group compared to control (puncta number data shown as mean ± s.e.m: fl/fl 3.49E7 ± 3.32E6, cKO 2.4E7 ± 1.37E6; Fig. 6L; VGLUT2 volumes shown as mean ± s.e.m. [volume µm³]: fl/fl 0.75 ± 0.01, cKO 0.66 ± 0.01; Fig. S6Q), as well as reduced VGLUT2-PSD95 synaptic marker colocalization (synapses; ~50%, fl/fl 8.6E6 ± 1.11E6, cKO 5.06E6 ± 6.55E5; Fig. 6N) with no change in PSD95 puncta number (fl/fl 1.11E8 ± 9.3E6, cKO 1.04E8 ± 5.87E6). Altogether, these results further support a central role for astrocytic store-released Ca^2+^ in regulating the development of glutamatergic synapses in the visual cortex.

## Discussion

This study reveals that astrocytic IP3R2-mediated Ca^2+^ signaling is essential for maturation and refinement of visual system synapses, circuits, and behavior. Specifically, we show:

- IP3R2 KO mice exhibit reduced numbers and volumes of presynaptic VGLUTs and VGLUT-containing synapses at P14 and P28, but not P7. These reductions are specific to glutamatergic presynaptic terminals, with no changes in the number of postsynaptic densities, global axonal innervation to the visual cortex, total cell number, or inhibitory GABAergic synapse number.
- Visually evoked neuronal c-FOS activation in response to a light pulse is reduced in IP3R2 KO mice across visual circuit brain regions, while baseline levels of neuronal c-FOS activity remain unaffected.
- Defensive behavioral responses (e.g., escape to shelter) to a visually triggered looming stimulus are diminished in IP3R2 KO mice despite normal ambulatory activity.
- Astrocyte morphogenesis is perturbed in the VC of IP3R2 KO mice at P16 during the period of peak synaptogenesis, concurrent with differential expression of cell-growth related genes and remains diminished at P30, with fewer structural synapses within KO astrocyte territories.
- Postnatal conditional knockout of astrocytic IP3R2 recapitulates deficits in VGLUT2-containing structural synapses in the P16 VC.

The appropriate spatio-temporal expression of synaptic proteins is critical for proper synapse maturation in the developing brain. In mice lacking IP3R2, we observed a developmentally specific reduction of presynaptic VGLUTs (Fig. 1, Fig. S1, Fig. S2, Fig. 6, Fig. S6), expanding on our previous findings demonstrating a dysregulation in the expression of post synaptic AMPA receptor subunits GLUA1 and GLUA2 in IP3R2 KO VC at P14^4^. Accompanied by our results that IP3R2 protein levels were stable across development (Fig. 1B), this suggests that IP3R2 signaling becomes increasingly important as synapses mature. While initial stages of synaptogenesis in the VC may be driven by neuron autonomous programs or astrocytic store-released Ca^2+^-independent mechanisms, later timepoints, such as the period following eye opening, likely require astrocytic input mediated by store-released Ca^2+^ signaling to stabilize synaptic connections. This aligns with previous work showing that astrocytic gene expression becomes increasingly specialized during specific stages of synapse development, and that Ca^2+^ dependent transcriptional regulation is a major mechanism by which astrocytes influence circuit assembly and responses to neuronal activity^4,12,30,37^.

Notably, synaptic deficits in IP3R2 KO mice were limited to glutamatergic circuits, with structural inhibitory synapses remaining largely intact, showing a mild but significant reduction in the presynaptic VGAT puncta volumes at P28 (Fig. 3). This potentially suggests impaired inhibitory function, which can also stem from reduced excitatory input onto interneurons. This has important implications for cortical excitatory/inhibitory (E/I) balance. Disruptions in E/I ratio are a hallmark of multiple neurological disorders^66–69^, and previous studies have linked astrocyte Ca^2+^ signaling to their pathogenesis. For example, it was shown that germline IP3R2 KO mice exhibit disrupted resting-state functional connectivity in medial prefrontal cortex (mPFC)-centered networks, mirroring patterns observed in humans with major depressive disorder^70^, which is characterized by disrupted glutamate and GABA signaling^6^. Early life disruptions in glutamatergic synapses caused by dysregulated astrocytic store-released Ca^2+^ signaling could precede the development of these disorders in the mature brain. Research focusing on characterizing the excitatory/ inhibitory neuronal activity (using methods such as electrophysiology or Ca^2+^imaging) as well as identifying the downstream Ca^2+^ dependent pathways in astrocytes that selectively influence excitatory synapse maturation and maintain E/I balance during critical developmental periods will be essential to improving our understanding of these processes.

At the circuit level, we found that evoked (but not basal) neuronal activation was impaired in IP3R2 KO mice. Expression of the immediate early gene c-FOS, which we found to be almost exclusively present in neurons and not astrocytes at P16^49,51^ was significantly reduced in response to visual light stimulation across both cortical and subcortical regions of the visual circuit including the VC, dLGN, and SC (Fig. 4, Fig. S4). These findings are consistent with previous reports demonstrating that astrocytic Ca^2+^ transients contribute to stimulus dependent neuronal activation and cortical state transitions, particularly in sensory cortices where astrocytic Ca^2+^ activity has been shown to gate sensory throughput and regulate network gain^42,71^. Specifically, IP3R2 KO was shown to elevate sensory evoked gamma activity, an effect that reflects reduced astrocyte-mediated modulation of cortical excitability and aligns with our observation of impaired neuronal activation following visual stimulation. Further, the suppression of visually evoked c-FOS expression in IP3R2 KO mice is consistent with work showing that astrocyte mediated modulation of excitatory neurotransmission is critical for experience-dependent plasticity in early postnatal visual circuits^72^. Future studies employing acute functional measurements such as Ca^2+^ activity or electrophysiology will reveal the specific contribution of IP3R2-mediated Ca^2+^ signaling to light-evoked neuronal responses in the developing visual circuit.

At the behavioral level, these circuit impairments translated into abrogated defensive responses to a visual looming threat and altered rearing behaviors (Fig. 5, Fig. S5). The defensive behavioral deficits may in part be caused by disruptions in evoked activity in both the visual cortex and specifically, the superior colliculus (Fig. 4), which is critical for the development and execution of defensive responses in mice and humans^73,74^. In addition to the visual component, anxiety and stress are known to influence defensive responses^75^. There was no difference between genotypes in time spent in the center versus periphery of the arena, suggesting similar anxiety levels. However, the observed increase in supported and decreased unsupported rearing may indicate an elevation in baseline stress levels^76,77^, though recent findings have decoupled this relationship^78^. Notably, heightened stress potentiates looming-evoked escape^75^, suggesting that the diminished defensive responses in IP3R2 KO mice may reflect an impairment upstream of the emotional state, potentially in visual threat detection or processing, rather than an anxiety driven suppression of escape. Future studies leveraging targeted circuit level manipulations will be necessary to determine whether these effects arise from retinal processing dysfunction (which was not assessed in the present study), alterations in top-down processing from the visual cortex, deficits intrinsic to the superior colliculus, or disruptions in stress and arousal linked pathways that modulate defensive responses. These findings further align with reports of disrupted social behaviors in adult IP3R2 KO mice, including delayed dominance behaviors, impaired social interaction, and autism spectrum disorder-like phenotypes in both germline and astrocyte specific IP3R2 KO models, underscoring the prominent astrocytic contributions to these behaviors^22,37^. Furthermore, it was shown that the onset latency of social dominance behavior, but not the overall behavior itself, was disrupted in IP3R2 KO mice^37^. Both defensive and social interactions require cortical integration of salient sensory cues to generate appropriate behavioral outputs. In accordance, we observed that defensive response types were differentially modulated in IP3R2 KO mice (Fig. 5). The selective loss of “Freeze+Escape” response type suggests a deficit in coupling sensory input with context-dependent behavioral output, or an impairment in the ability of IP3R2 KO mice to transition from one behavioral state to the next, which may contribute to social impairments identified in other studies^22,37^. Together, these observations suggest a potential common mechanism in which disrupted astrocytic Ca^2+^ signaling compromises the integration of sensory information with appropriate behavioral outcomes.

Such selective disruptions may also help reconcile why some studies have reported no discernable changes in synaptic activity or behavior in IP3R2 KO mice^26,39^. Behavioral outputs are highly modular in nature, with specific astrocytic events contributing to different components of a behavior, such as network tuning required for it to begin or end. Thus, while IP3R2-mediated store-released Ca^2+^ is probably not responsible for every aspect of the behavioral outputs that it is involved in, it likely plays important roles in shaping their specific components and temporal dynamics, an idea consistent with recent reports implying that astrocytic Ca^2+^ is important to behavioral state transitions^79–82^. Moreover, some astrocytic Ca^2+^ linked behaviors occur entirely independent of IP3R2 mediated signaling^31^, emphasizing a need for studies that quantify how different types of astrocytic Ca^2+^ signals integrate to produce functional outputs^17^. Future specified studies will be necessary to determine how astrocytic Ca^2+^ signaling interfaces with neuromodulatory systems and whether restoring evoked responses in IP3R2 deficient circuits can rescue behavioral deficits.

A major unresolved question involves the potential mechanisms by which astrocytes may induce their regulatory effects on synaptic maturation, and the role that store-released Ca^2+^ plays in this function. We approached this question by assessing gene expression changes in IP3R2 KO mice and identified broad dysregulation in cell growth and metabolism related genes. We further quantified astrocytic morphogenesis at P16 and P30, observing reduced volume and area of VC KO astrocytes (Fig. 6, Fig. S6), suggesting a possible mechanistic link to the observed developmental and functional phenotypes. This aligns with studies showing that Ca^2+^ dependent pathways are relevant to astrocyte growth and structural maturation^83,84^. Appropriate outgrowth and process elaboration is critical for proper synaptic ensheathment and gliotransmitter release, and loss or reduction of these functional processes could underlie deficits in astrocyte-neuron signaling, thus impairing synaptic integrity. Indeed, we observed fewer numbers of synaptic puncta within the territories of astrocytes lacking IP3R2 (Fig. 6, S6). Yet, whether these reductions are secondary to the morphological deficits in astrocyte volume and territory elaboration or represent an additional cell-autonomous impairment in the astrocyte-dependent mechanisms that promote glutamatergic synapse formation and maintenance remains to be determined. Interestingly, while overall volume and surface area were reduced in IP3R2 KOs, the geometric parameters of astrocyte shape such as sphericity and ellipticity were preserved. This suggests that IP3R2-mediated Ca^2+^ signaling selectively regulates growth rather than global cytoskeletal patterning mechanisms. Future work will determine whether these correlative morphological deficits disrupt functional astrocyte–synapse interactions in vivo and how they relate mechanistically to gliotransmission or synapse specificity.

In summary, in the present study we demonstrate that IP3R2 mediated Ca^2+^ signaling is an essential intermediary in the development of excitatory synapses in the mouse visual system. Given that astrocytic Ca^2+^ signaling is highly heterogeneous spatially, temporally, and mechanistically^15,32,33,35,40,85–89^, our findings provide direct evidence for the contribution of a defined signaling pathway (ER store-released Ca^2+^ via IP3R2) within a specific circuit (visual circuit), and during a critical developmental period (P14-P30). By establishing a role for IP3R2 mediated Ca^2+^ signaling in excitatory synapse maturation and visually driven behaviors, this work advances current understanding of how astrocyte-neuron interactions shape circuit development and maturation. Of note, though this work was mostly completed in a germline IP3R2 KO model, the glutamatergic synaptic findings were replicated in astrocyte-specific conditional KO animals, supporting the astrocyte-centric role of IP3R2 in the developing visual system. This is further corroborated by the significant enrichment of IP3R2 in astrocytes over neurons in our IHC data (Fig. 1) and previous studies^26,27^, however, we cannot completely rule out contribution of other cell types to all the observed effects. Further research on the functional implications of astrocyte specific loss of IP3R2 within select brain regions will be important to understanding the mechanisms by which this signaling pathway modulates brain dynamics. Overall, these findings underscore the need to better understand how astrocytes integrate diverse Ca^2+^ signals to coordinate their functions across circuits. Disentangling these complex relationships necessitates studies combining astrocyte specific manipulations, in vivo imaging, and computational modeling will be essential to our understanding of how distinct astrocytic Ca^2+^ signals contribute to synapse development, circuit function, and behavior.

## Methods

### Animals

All animal work was approved by the Texas A&M University (TAMU) Institutional Animal Care and use Committee (IACUC) (Animal Use Protocol #2023-0244). We have complied with all relevant ethical regulations for animal use.

Mice were maintained under standard housing conditions on a 12-h light:dark cycle with *ad libitum* access to food and water. Both female and male mice from newborn through adult were used in experiments. Mice were allocated to the experimental groups based on genotype/treatment, sex, and age.

The following mouse lines were used: Wild type (WT; C57Bl6/J) were purchased from Jackson Labs and bred in-house (Jax #000664). Mice were used for breeding and backcrossing, and as controls. IP3R2 KO (Itpr2^tm1.1Chen^) was originally obtained from the Chen lab^65^ and maintained on C57BL6/J background as KO X KO or het X het breeding schemes. To generate experimental groups, KO mice were compared with WT collected at the same developmental stages and time-of-day. tdTomato reporter B6.Cg-Gt(ROSA)26Sor^tm14(CAG-tdTomato)Hze^/J (Ai14; Jax #007914) were purchased from Jackson Labs and maintained in-house. Mice at P1-2 were injected ICV with GfaABC1D-cre AAV to induce recombination and tdTomato reporter expression. IP3R2 flox (Itpr2^tm1Chen^)^65^ were obtained from Sur lab (MIT). Mice at P1-2 were injected ICV with AAVs to express cre recombinase (or control scrambled peptide) under minimal GFAP promoter (GfaABC1D) for astrocyte-specific IP3R2 knockout.

### Mouse tissue collection

#### Histology

Tissue was collected at post-natal days (P) 7, 14-16, 28-30. Mice were anaesthetized by IP injection of 100 mg/kg Ketamine /20 mg/kg Xylazine mix (obtained from Comparative Medicine program at TAMU) and transcardially perfused with PBS, then 4% PFA at room temperature. Brains were removed and incubated in 4% PFA overnight at 4 C, then washed 3 × 5 min with PBS, cryoprotected in 30% sucrose for 2–3 days, then embedded in TFM media (VWR # 100496-345), frozen in dry ice-ethanol slurry solution, and stored at –80 °C until use. Brains were sectioned using a cryostat (Leica CM1950) in sagittal or coronal orientations depending on experimental needs at a slice thickness of 18–20 µm, or 100 µm. Sections were either mounted on Superfrost plus slides (Fisher #1255015) or kept in PBS (as floating sections) and either used immediately for histological procedures (Immunohistochemistry; IHC) or stored at –80°C /4 C for later use. 3–5 mice from each sex and age group were used unless stated otherwise in Figure legends. For each mouse, 2–3 sections were imaged and analyzed.

#### Western Blot /qPCR

Mice were anaesthetized by IP injection of 100 mg/kg Ketamine /20 mg/kg Xylazine mix and then decapitated. Brains were rapidly removed and the VC dissected in ice-cold PBS, flash frozen and kept at –80 °C until use. 3–5 samples for each condition were analyzed.

### Immunohistochemistry

#### Slide mounted sections

Sections were blocked for 1 h at room temperature in blocking buffer containing antibody buffer (100 mM L-lysine and 0.3% Triton X-100 in PBS) supplemented with 10% heat-inactivated normal goat serum (Gibco # 16210072). Primary antibodies diluted in antibody buffer with 5% goat serum were incubated shaking overnight at 4 °C. The following day, sections were washed 3 × 5 min with PBS with 0.2% Triton X-100 and secondary antibodies conjugated to Alexa Fluor were applied for 2 h at room temperature. Slides were mounted in SlowFade Gold media with DAPI (LifeTech #S36939), covered with #1.5 glass coverslips (Fisher #12544E), and sealed with clear nail polish.

#### Free-floating sections

Sections were blocked for 1 h shaking at room temperature in blocking buffer containing antibody buffer (100 mM L-lysine and 0.3% Triton X-100 in PBS) supplemented with 10% heat-inactivated normal goat serum. Primary antibodies diluted in antibody buffer with 5% goat serum were incubated shaking for 24 h at 4 °C. The following day, sections were washed 3 × 15 min with PBS with 0.2% Triton X-100 and secondary antibodies conjugated to Alexa Fluor were applied for 2 h shaking at room temperature. Slides were mounted in SlowFade Gold media with DAPI (LifeTech #S36939), covered with #1.5 glass coverslips (Fisher #12544E), and sealed with clear nail polish.

The following primary antibodies were used: Rb anti IP3R2 (Alomone labs #ACC-116, 1:250), Gp anti-VGLUT1 (Millipore #AB5905, 1:1000), Gp anti-VGLUT2 (Millipore #AB2251, 1:1000), Rb anti-PSD95 (Thermo-Fisher #516900, 1:250), Gp anti-VGAT (Synaptic Systems #131004, 1:250), Rb anti-Gephyrin (Synaptic Systems #147008, 1:500), Rb anti-NF200 (Millipore Sigma #N4142, 1:400), Chk anti-GFP (Invitrogen A10262, 1:1000), Rb anti-S100β (Abcam #AB52642, 1:100), Gp anti-S100β (Synaptic Systems # 287004, 1:200), Gp anti-c-FOS (Synaptic Systems # 226308, 1:1000), Ms anti-NeuN (Millipore #MAB377, 1:100). The following secondary antibodies were used: Gt anti-Rb Alexa-488 (Invitrogen #A11043), Gt anti-Gp Alexa-488 (Invitrogen # A11073), Gt anti-Gp Alexa-555 (Invitrogen #A21435), Gt anti-Chk Alexa-488 (Invitrogen #A32931), Gt anti-Rb Alexa-594 (Invitrogen #A11037), Gt anti-Gp Alexa-594 (Invitrogen #A11076), Gt anti-Ms Alexa-647 (Invitrogen #A21236), Gt anti-Gp Alexa-647 (Invitrogen #A21450). All secondary antibodies were used at 1:500 dilution.

### Microscopy and imaging

#### Fluorescent microscopy

Fluorescent microscopy was performed to image the expression of IP3R2 in cortical layers (Fig. 1, Fig. S1), developmental changes in NF200 (Fig. 2, Fig. S3), and c-FOS expression (Fig. 4, Fig. S4) using a Leica THUNDER Imager with LED3 light source, and sCMOS camera (Leica DFC9000) at ×20 magnification. Single plane images (2048 × 2048 pixels; 665.49 × 665.49 µm) containing the visual cortex or hippocampus (for NF200) were taken for IP3R2 validation and NF200 studies (Fig.1, Fig. S1, Fig. 2, Fig. S3). For c-FOS imaging experiments (Fig. 4, Fig. S4), z stack images (2048 × 2048 pixels; 665.49 ×  665.49 × 6 µm) containing the visual cortex, hippocampus, dorsal lateral geniculate nucleus, or superior colliculus were acquired for each brain section separately. A tile image was taken to encompass all visual cortex neuronal layers (2 tiles). Example images are shown as a single z plane image from the same location in the stack for each genotype. Thunder processing (Leica LASX software) was performed using default parameters for single plane imaging (Instant Computational Clearing) to increase resolution and image clarity in the same way for all images.

#### Confocal microscopy

Confocal microscopy was used to image IP3R2 expression, synaptic proteins, TdTomato expression, and Lck-eGFP labeled astrocytes (Fig. 1, Fig. S1, Fig. S2, Fig. 3, Fig. 6, Fig. S6). Imaging was performed using a Zeiss LSM 900 upright confocal laser scanning microscope with Airyscan2. IP3R2 imaging (Fig. 1, Fig. S1, Fig. S6) was acquired at ×63 magnification as single plane images (2384 × 2384 pixels; 101.41 × 101.41 µm). Imaging of VGLUT/PSD95 and VGAT/Gephyrin puncta (Fig.1, Fig. S2, Fig. 3) were acquired at ×63 magnification. For each section, a 1024 × 1024 pixels (101.4 × 101.4 × 2.79 µm) z-stack image was obtained (pixel size 0.09 × 0.09 × 0.31 µm; 10 slices per 2.79 µm). Imaging of astrocyte morphology (Fig. 6) was acquired at ×63 magnification using the Airyscan2 module as a 1834 × 1834 pixels (78.01 × 78.01 XY µm), and 27–47 µm z-stack to ensure images included the entire astrocyte, (pixel size 0.043 × 0.043 × 0.15 µm; Fig. 6D, E). For imaging of synapses within astrocyte territory (Fig. 6, Fig. S6), a 3 µm z-stack (21 slices) using the parameters described for astrocyte morphology was centered around the largest part of astrocyte territory in XY plane (Fig. 6F, S6H). For validation of Lck-eGFP and tdTomato colocalization with astrocyte marker S100β (Fig. S6), 20× magnification was used to acquire 512 X 512 pixels (247.35 × 247.35 × 15 µm) z-stacks (pixel size 0.48 × 0.48 × 0.31 µm). Example images of IP3R2 signal and synaptic proteins with or without co-labeled astrocytic Lck-eGFP (Fig. 1, Fig. S1, Fig. S2, Fig. 3, Fig. 6. Fig. S6) are shown as a single z plane image from the same location of the stack for each genotype. Example images for astrocyte morphology (Fig. 6, Fig. S6) and 20× images of Lck-eGFP and TdTomato colocalized with S100β are shown as maximum intensity projections.

### Image analysis and quantification

Image analysis was done with ImageJ (NIH) or Imaris (Bitplane) software as described:

Quantification of pre /postsynaptic puncta and synapses - (Figs. 1, 3, 6) was performed as previously described^4^ on 3D images using Imaris software (Bitplane). The “surfaces” function was used to create volumes for VGLUT or VGAT signals, while the “spots” function was used to render PSD95 or Gephyrin. Total number of surfaces/spots was obtained for pre- and post- synaptic puncta count, and synapses were quantified by counting post-synaptic “spots” located within ≤0.2 µm of a presynaptic “surface”. The number of total and colocalized puncta was compared between the experimental groups. A minimum of three sections per mouse were imaged and analyzed for each brain region, and the experiment was repeated in at least five WT and KO pairs. Volumes were computed from the surfaces generated for VGLUT1, VGLUT2, or VGAT and exported from Imaris.

Quantification of IP3R2 signal (20× and 63× imaging) - (Fig. 1, Fig. S1) Images labeled with IP3R2 and S100β (to label astrocytes) or Neun (to label neurons) were analyzed using a semi-automated custom-made macro in ImageJ^4^. For layer specific analysis (20×) cortical layers (L1, L2-4, L5-6) of each image were manually cropped and saved to a new file. High resolution 63× confocal images (Fig. 1, Fig. S1, Fig. S6) were analyzed either as a single plane image (Fig. 1, Fig. S1), or maximal intensity projections (Fig. S6). For each of the different signals (IP3R2, S100β or Neun), images were manually thresholded in the same way for each section, and the IP3R2 signal area within S100β or Neun ROIs, or total (not marker-colocalized signal) was calculated as thresholded area. We utilize thresholded area as our preferred signal quantification over intensity parameters (such as mean gray value or integrated density) as intensity parameters tend to be more variable across multiple experiments due to differences in signal intensity, reducing the robustness of the analysis. Resulting thresholded areas of the IP3R2 signal were normalized to the total area to obtain thresholded area per mm^2^ (for 20× images) or to the number of cells within the image to obtain signal per cell in µm (for 63× confocal images).

Quantification of axonal density - (Fig. 2, Fig. S3) was performed in two ways: (1) quantification of NF200 fluorescent signal (as thresholded area), and (2) quantification of axonal fragments as skeletons. For NF200 fluorescent signal analysis, images labeled with anti-NF200 antibody (to label axons) and DAPI (to label nuclei) were analyzed using a semi-automated custom-made macro in ImageJ. For each image, VCs were manually cropped and saved to a new file. Images were thresholded in the same way for each section for NF200 signal, and the “Analyze Particles” function was used with a size range of 10–150 pixels to quantify DAPI. Resulting average thresholded area was normalized to the total area analyzed to obtain thresholded area per mm^2^, and cell counts were normalized to the total area to obtain the number of cells per mm^2^. For skeletonized analysis, cortical layers 1 and 2–4 were manually cropped and saved as single-channel (grayscale) TIFF files. Images were then converted to a binary format and processed with the ImageJ “Skeletonize” plugin to obtain single-pixel-wide centerlines representing the medial axis of each axonal fragment, then analyzed with the “Analyze Skeleton” plugin to output axonal fragment length and density metrics.

Quantification of c-FOS - (Fig. 4, Fig. S4) Images labeled with anti-c-FOS antibody and DAPI (to label nuclei) were analyzed using a semi-automated custom-made macro in ImageJ. Maximum intensity projection images were generated from z stacks, cropped by cortical layer (VC) or region (dLGN, SC, CA1, DG), and saved as individual files for analysis. Sections containing dLGN were co-stained for VGLUT2 to identify regional boundaries. Each channel was thresholded manually in the same way for each image. For c-FOS, particles within a size range of 25–350 pixels were detected, and the total count within ROIs was obtained. For DAPI, particles sized 10–150 pixels were segmented following watershed separation and counted. Cell counts were normalized to the total area to obtain the number of cells per mm^2^. To quantify c-FOS colocalization with neuronal and astrocytic cell markers, analysis was performed in two ways: (1) thresholding analysis of the VC using custom-made semi-automated macro in ImageJ (Fig. S4H; similar to the approach used to quantify IP3R2 signal within cortical layers, Fig. S1). For each image, VCs were manually cropped and saved to a new file. Images were thresholded in the same way for each section, and the number of c-FOS positive cells colocalized with each of the cell type specific markers (Neun for neurons, S100β for astrocytes) was obtained and normalized to total area to obtain the number of cells per mm^2^, both the total number of each cell type and those co-labeled with c-FOS. (2) manual cell counting within defined ROIs in the VC (0.06 mm^2^) using the cell counter plugin in imageJ. For each image, and ROI encompassing layers 2–4 of the VC (representing the majority of c-FOS signal in the upper region of the VC) was manually cropped and saved to a new file. Each image was then manually counted Neun, S100β, and c-FOS colocalized cells to obtain cell number within 0.06 mm^2^ (Fig. S4G). This data was also used to calculate percentage of c-FOS cells colocalizing with each of the cell markers (Fig. S4I).

Quantification of astrocyte morphology - (Fig. 6, Fig. S6) was performed on 3D images using Imaris. The “surface” function was used to create volumes using the absolute intensity filter and cropped to remove partial signal from neighboring astrocytes within the field of view. Remaining surfaces were unified to generate outputs for total volume and area as well as sphericity and ellipticity. A minimum of 5 cells from 3 different sections in at least 5 WT and KO pairs were imaged and analyzed.

Quantification of GfaABC1D-Lck-GFP or tdTomato colocalization with S100β - (Fig. S6) Images containing astrocytes expressing Lck-eGFP (Fig. S6C) or tdTomato (Fig. S6L) were co-labeled with antibody for S100β (to mark astrocytes) and analyzed using a semi-automated custom-made macro in ImageJ. For each of the different signals (Lck-eGFP, tdTomato, S100β), images were thresholded in the same way for each section, and the “Cell Counter” plugin in imageJ was used for manual count of each of the cell types, as well as the colocalized cells. Resulting cell and colocalization counts were summarized to obtain percent (%) overlapping cells.

### Adeno associated viral (AAV) vectors and intracerebroventricular (ICV) injection

ICV injection was performed as previously described^90–92^. P1-2 mouse pups were removed from their home cage and placed in a warmed holding cage. Pups were individually cryo-anaesthetized in ice for 5 min (placed inside a glove) before being transferred to a surgical pad over a back-lit surface. The ventricles were identified by measuring the distance 2/5^ths^ from the lambda suture to the eye. 1–2 µL of AAV5 particles, diluted to 2 × 10^9^ – 1 × 10^12^ gc/mL in saline was drawn into the barrel of a 10 µl glass syringe (Hamilton #7653-01) fitted with a 32 G injection needle (Hamilton #7803-04). With the pup’s head upright and secured by hand, the injection site was swabbed with 70% ethanol, and the tip of the needle was positioned perpendicular to the injection site and inserted ~3 mm before the plunger was manually depressed. The syringe was held in place for 10 s to avoid ejection, and then carefully withdrawn. This procedure was performed for both ventricles. After injection, pups were returned to the warmed holding cage for immediate recovery, then to their home cage for two-four weeks before brain tissue collection. AAV constructs were cloned by VectorBuilder and purchased as packaged viral particles, which were aliquoted and stored at –80 °C until use. AAVs used: pAAV[Exp]-GFAP(short)>Lck-GFP:IRES:ORF Stuffer: WPRE (VB240226-1200bvh); pAAV-GfaABC1D-EcoRI/(Lck-GFP):IRES:ORF_Stuffer/NdeI-4x6T (VB250512-1483qjy); pAAV-GfaABC1D-EcoRI/(Lck-GFP):IRES:Cre/NdeI-4x6T (VB010000-9287ffw).

### Light pulse assay

P16 mice (aged to ensure eyes were open for at least 24 h before light pulse experiments) were habituated in cages placed within darkened boxes (32 W × 38 L × 32 × 54.6H cm) for 30 min prior to experimentation, then mice were moved (individually) to either a control cage within a darkened box, or an experimental cage in a separate box fitted with 3 white LED light strips (110 lumen each). Experimental mice were subjected to 330 lumen ( ~ 2713 lux) light for 20 min, followed by immediate anesthesia by Ketamine/Xylazine injection and perfusion as described (see Mouse tissue collection section). Control mice remained in dark condition for 20 min before tissue collection. The light pulse procedure was performed for all mice between ZT16 and ZT17 after four hours of sustained darkness (ZT12-ZT16) under standard housing conditions.

### Behavioral analysis

The visually triggered defensive behavior assay was performed according to our previously published protocol^58^ modified from^59^. Briefly, the setup consists of a rectangular arena (17 W × 40 L × 20H cm) with a plastic shelter(11 cm in diameter, 5 cm height) placed in one corner of the cage. The shelter used is identical to that used in the mouse home cages to facilitate quicker familiarization and habituation to the testing environment. A computer monitor is mounted 40 cm above the arena floor, connected to a computer through which the looming stimulus is delivered. A video camera is placed above the cage to record mouse movement during the stimulus for offline analysis. The test was administered over 3 consecutive days and consisted of a habituation day (day 0), followed by 2 consecutive testing days (see Fig. 5A). During habituation, mice are placed in the testing arena one at a time, and allowed to explore for 13 min, without the looming stimulus played. On each testing day, mice are placed in the arena and subjected to 5 trials of the looming stimulus. The first stimulus is played after a minimum of 3 min of free exploration, with a minimum of 1 min interval between subsequent stimuli, during which time mice engage in exploratory behavior. Mice spend up to 13 min in the arena on testing days, then are returned to home cages even if they did not receive 5 trials. Stimulus is played only if a mouse is outside the shelter and engaged in exploratory behavior. Day 2 is identical to Day 1, occurring ~24 h later. Mice are returned to the vivarium between testing days.

The looming stimulus - is a 9 s video showing an expanding black disc (2–20 cm in diameter) on a white background is played to mimic a looming threat (file available from^59^; see Fig. 5B). The stimulus begins when a black disc (2 cm) appears on the computer screen ( ~ 3.5 s), rapidly expands to 20 cm diameter ( ~ 2 s) and remains fully expanded for ~3.5 s. This stimulus has been thoroughly characterized in previous studies, including our recently published work, to elicit robust defensive responses in mice^56,58,59^.

Defensive behavioral scoring - on each testing day, three types of defensive responses are recorded during stimulus presentation, and manually scored by the experimenter either live during testing, or offline from recorded videos, as 1—response, 0—no response (i.e., mouse continues exploratory behavior or movement with no directed change due to stimulus): “Freeze”—a mouse’s snout and body are immobile for a minimum of 1 s; “Escape”—the mouse is moving towards and entering the shelter; “Freeze+Escape”—a period of immobility followed by movement and entering the shelter. In addition, two types of rearing behaviors were quantified both during stimulus and during the entire testing period: “Supported rearing” – was quantified using BehaviorDEPOT’s rearing classifier (v1.6^93^) to obtain a list of rearing events per mouse. An Arena_Floor ROI was drawn on the first frame of each video to delimit the planar floor of the open field, inset from the arena walls. A rearing event was counted if the tracked body part (mid back) shifted outside the Arena_Floor ROI (the planar floor of the arena) for longer than 0.5 s. “Unsupported rearing”—was manually scored from recorded videos using the Chronotate software^94^, to obtain list of rearing events per time frame. A rearing bout^57,76,77^ was counted if the mouse was observed standing on its hind paws without support from the arena wall or any object (such as the shelter), with front paws detached from the ground and raised to chest level for a minimal duration of 0.5–1 s.

Kinematics analysis, locomotion and arena localization - was done using DeepLabCut^95,96^ trained custom pose estimation model, which was applied to the video recordings to generate X-Y coordinates for each frame. The resulting data was used as input by BehaviorDEPOT^93^ to calculate the velocities (computed using the Pythagorean Theorem: √[(dx/dt)² + (dy/dt)²]), total distance traveled, and percent time spent in center vs periphery of the arena. Custom MATLAB and Python scripts were used to extract kinematic data from the entire recording and from specific frame ranges corresponding to the presentation of the looming stimulus, respectively (Fig. 5, Fig. S5). For open field analysis (arena center vs periphery localization), two regions of interest were drawn manually on the first frame of each video: an Arena ROI encompassing the full open field, and a Center ROI demarcating the central zone. For each ROI (Center, Periphery), total time spent was extracted for each animal and used to calculate percentage of time spent out of total time.

Exclusion criteria- mice that missed more than 2 stimuli (e.g., remaining inside the shelter/failure to explore) on either testing day were removed from the study.

### Western blot

Samples were heated in reducing sample buffer (Thermo #39000) for 45 min at 55 °C. For tissue lysates, 5 µg/lane was loaded. Samples were resolved on Bolt 4–12% Bis-Tris gels (Invitrogen #NW04125BOX) for 1 h and 15 min at 150 V. Proteins were transferred to Immobilon-FL PVDF membranes (Millipore #IPFL00010) at 20 V for 1 hr, then blocked in 1% casein (Bio-Rad #1610782) in TBS (Bioworld #10530027-2) blocking buffer for 1 h at room temperature on a shaker. Primary antibodies were applied overnight at 4 °C, diluted in blocking buffer. The primary antibodies used were: Rb anti-IP3R2 (Alomone labs # ACC-116, 1:500), and Ms anti-GAPDH (Cell Signaling Technology #97166 T, 1:2000). The next day, membranes were washed 3 × 10 min with TBS-0.1% Tween (Promega #PRH5152) and secondary antibodies Gt anti-Rb Alexa-680 (Invitrogen #A21109) and Gt anti-Ms-Alexa-800 (Invitrogen #PIA32730) were applied for 2 hr at room temperature (dilution 1:10,000). Bands were visualized using the LI-COR Odyssey CLx Infrared Imager and band intensity analyzed using the LI-COR Image Studio software.

### Quantitative real-time PCR (qPCR)

Total RNA was isolated from 15-20 mg of flash-frozen cortical tissue using RNeasy Plus Mini Kit (Qiagen #74136). RNA yield and purity was analyzed by NanoDrop ND-1000 spectrophotometer (Nanodrop technologies, USA), and first-strand cDNA was synthesized from 1 μg total RNA using SuperScript VILO MasterMix (Invitrogen #11755-050) following the manufacturer’s instructions. Quantitative real time PCR was executed using a Bio-Rad CFX Opus 96 Real-Time PCR System (Bio-Rad, USA). For each reaction the 20 μl reaction mixture contained 1 μl cDNA template, 8 μl molecular biology grade water, 1 μl primer mix (forward and reverse, 10 μM), and 10 μl 2X SYBR Green PCR Master Mix (Applied Biosystems #4364344). RNA levels were quantified for relative expression using the ΔCT method with *Gapdh* as a reference transcript. PCR reactions were performed in triplicate for each cDNA sample. Primers were designed from nucleotide sequences obtained using NCBI BLAST and synthesized by Integrated DNA Technologies. Primer pairs used are as follows: GAPDH-F CATCACTGCCACCCAGAAGACTG, GAPDH-R ATGCCAGTGAGCTTCCCGTTCAG; IP3R2-F AGACTGCCTGTTCAAGGTGTG, IP3R2-R ATGGTTCCCCTGTTTCGCCTG; CST3-F CATACAGGTGGTCAGAGCTCGT, CST3-R CTGGTCATGGAAAGGACAGTCAG; FSTL1-F CCACCTTCGCCTCTAACTCG, FSTL1-R ATCGTTTCCACATCGTGCTC; GLUD1-F TCCGTTACAGCACTGACGTGAG, GLUD1-R ACGCCTGCTTTAGCACTGCCAA; GRIN2B-F CTGGTGACCAATGGCAAGCATG, GRIN2B-R GGCACAGAGAAGTCAACCACCT; KCNK2-F GCTATACTGCAGGAGTGGCG, KCNK2-R AGGAGAATGAGAGCCTCGGT; LGR4-F GTGCATTTTGGGGGTGTGAC, LGR4-R TGCTGCATCTGTAGCACCTTT; MAPK10-F AGCTGGATGAAAGGGAGCAC, MAPK10-R ACTGCTGCACCTGAAGGC; OGT-F GGCTATGTGAGTTCTGACTTCGG, OGT-R GATTGGCTTCCGCCATCACCTT.

### Statistics and reproducibility

Data is shown as mean ± s.e.m, median with range, or percentages, as indicated in each Figure legend. Descriptive statistics are reported in the text as 2 or more numbers after the decimal point unrounded. Statistical analysis was performed using Prism (Graphpad) software or R. Two-way ANOVA was used for multivariate data (such as effects of age and genotype on synapse development, Fig. 1, or effect of light pulse and genotype on c-FOS expression, Fig. 4) with multiple comparison correction by the two-stage linear step-up procedure of Benjamini, Kriger, and Yekutieli. Pairwise comparisons were done by *t* test (normal distribution) or Mann–Whitney rank sum test (non-normal distribution). Each dataset was tested for normality (with Shapiro-Wilks test) to ensure the correct statistical test is used. Fisher’s exact test was used to compare percentages of responding mice (Fig. 5, Fig. S5). Two sample Kolmogorov-Smirnov test was used to compare between cumulative frequency distributions (Fig. S2, Fig. 3, Fig. S6). Analysis was completed blind to genotype when possible. Sample sizes were determined based on our previous work^4,58^ and similar published studies^97–99^ to achieve adequate statistical power ( > 80%, α = 0.05) for detecting expected effect sizes (30-60%) while minimizing the number of animals used. Sample sizes, statistical tests used, and significance values are presented in each Figure and Figure legend. *P* value ≤ 0.05 was considered statistically significant. All the data including the input values (descriptive statistics) and full statistical analysis information (including all pairwise and multiple comparisons, ANOVA source of variation values etc.) are detailed in the document Supplementary Data 1 for each Figure as labeled.

### Reporting summary

Further information on research design is available in the Nature Portfolio Reporting Summary linked to this article.

## Supplementary information

Supplementary Information

Description of Additional Supplementary Files

Supplementary Data 1

Reporting Summary

## Data Availability

The numerical data supporting the findings of this study and full statistical analysis information are available within the paper and its supplementary information in Supplementary Data 1. Raw or unprocessed data (such as microscopy imaging files) is available upon request from the corresponding author, Isabella Farhy-Tselnicker (ifarhy@bio.tamu.edu).

## Code availability

The scripts used for analysis of behavioral data (GitHub_scripts) and the DeepLabCut model (DLC model) are available in the appropriate links. Code is also available through the zenodo repository https://doi.org/10.5281/zenodo.20650998.
