# Supplementary material for: Astrocyte store released calcium modulates structural synapse development and circuit output in the visual system
Source: Commun Biol. 2026 Jul 11;9:1456. doi: 10.1038/s42003-026-10579-0 (PMC13737306; doi:10.1038/s42003-026-10579-0)
Supplement: Supplementary file 1 [file 42003_2026_10579_MOESM1_ESM.pdf]

Supplementary Information

Astrocyte Store Released Calcium Modulates Structural Synapse Development and Circuit Output in the Visual System

Gillian Imrie, Jordan Mar, Madison B. Gray, Tatiana Quiceno, Isabella Farhy-Tselnicker

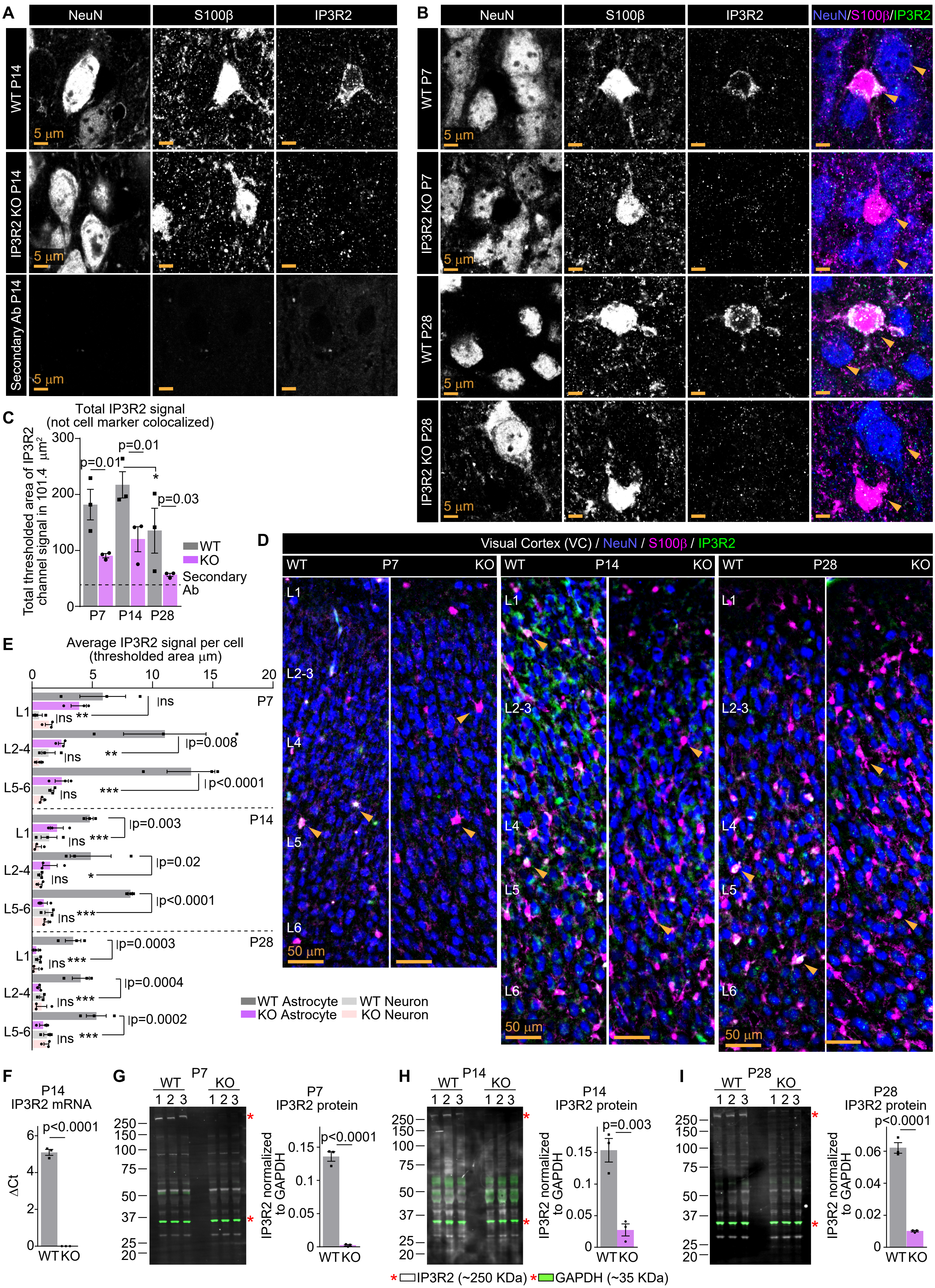

A
NeuN
S100β
IP3R2
WT P14
IP3R2 KO P14
Secondary Ab P14
5 µm
B
NeuN/S100β/IP3R2
WT P7
IP3R2 KO P7
WT P28
IP3R2 KO P28
C
Total IP3R2 signal (not cell marker colocalized)
Total thresholded area of IP3R2 channel signal in 101.4 µm²
p=0.01
p=0.01
p=0.03
WT
KO
Secondary Ab
P7 P14 P28
D
Visual Cortex (VC) / NeuN / S100β / IP3R2
WT P7 KO
WT P14 KO
WT P28 KO
L1
L2-3
L4
L5
L6
50 µm
E
Average IP3R2 signal per cell (thresholded area µm)
P7
P14
P28
L1
L2-4
L5-6
ns
p=0.008
p<0.0001
p=0.003
p=0.02
p=0.0003
p=0.0004
p=0.0002
WT Astrocyte
WT Neuron
KO Astrocyte
KO Neuron
F
P14 IP3R2 mRNA
ΔCt
p<0.0001
WT KO
G
P7
WT KO
1 2 3 1 2 3
P7 IP3R2 protein
IP3R2 normalized to GAPDH
p<0.0001
H
P14
P14 IP3R2 protein
p=0.003
I
P28
P28 IP3R2 protein
p<0.0001
* IP3R2 (~250 KDa) * GAPDH (~35 KDa)

Figure S1. Glutamatergic synapse development is perturbed in IP3R2 KO mice VC; related to Figure 1. See also Supplementary Data 1. **A.** Example single channel grayscale high power confocal images of P14 VC in WT and KO mice as labeled showing Neun, S100β, and IP3R2 signal (merged image is shown in Fig. 1B), as well as a secondary antibody (Ab) control showing background signal for each channel (bottom panel). **B.** Example high power confocal images of Neun, S100β and IP3R2 in WT and KO VC at P7 and P28 as labeled. Single channel grayscale images are on the left, merged images on the right. **C.** Quantification of total IP3R2 signal (from images in Fig. 1B, Fig. S1A-B; obtained by quantifying the IP3R2 signal without cell type markers colocalization), showing significant reduction of IP3R2 signal in KO mice at all developmental stages as labeled. Dashed line indicates average signal measured in the IP3R2 channel in the absence of primary antibody (secondary Ab control). **D-E.** IP3R2 signal is colocalized with S100β and reduced in the KO across neuronal layers in the VC. Example low power (20X) images of the WT and KO VC at the developmental stages as labeled. IP3R2 signal (green), S100β (magenta), Neun (blue) are shown. IP3R2 colocalized with the S100β signal appears as white color. **E.** Quantification of D, average of IP3R2 signal per cell within different layers (L1 – layer 1, L2-4 – upper layers, L5-6 – deep layers). **F.** Quantification of qPCR represented as change in IP3R2 Ct relative to *Gapdh* (ΔCT). IP3R2 mRNA was not detectable in IP3R2 KO mice tissue. **G-I.** Uncropped Western Blot images showing IP3R2 signal (white; imaged at 700 channel; see Methods) and GAPDH signal (green; imaged at 800 channel) for each age and genotype as labeled. Numbers above each lane indicate individual mouse samples. For each blot, quantification is shown on the right, represented as IP3R2 signal normalized to GAPDH loading control for each age and genotype as labeled. IP3R2 signal is strongly reduced in IP3R2 KO mice at all developmental stages. Graphs show mean ± s.e.m. Individual mouse data points are shown as circles or squares. Number of mice per group (N) = 3. Scale bar = 5 μm in A-B, 50 μm in D. Arrowheads indicate S100β and Neun positive cells in A-B, and S100β positive cells colocalized with IP3R2 in D. In C, E statistical analysis by two-way ANOVA with multiple comparison correction by the two-stage linear step-up procedure of Benjamini, Kriger and Yekutieli examining the effects of age and genotype (C), and cell type and genotype (E). *P≤0.05; **P<0.01; ***P<0.001 comparing age/cell type groups within each genotype; P value (p) on the plots comparing WT and KO groups. In F-I, P values (p) on the plots indicate comparison between WT and KO groups by t-test. ns denotes non-significant (P>0.05) results. Descriptive statistics and detailed statistical test results for all data panels are provided in Supplementary Data 1.

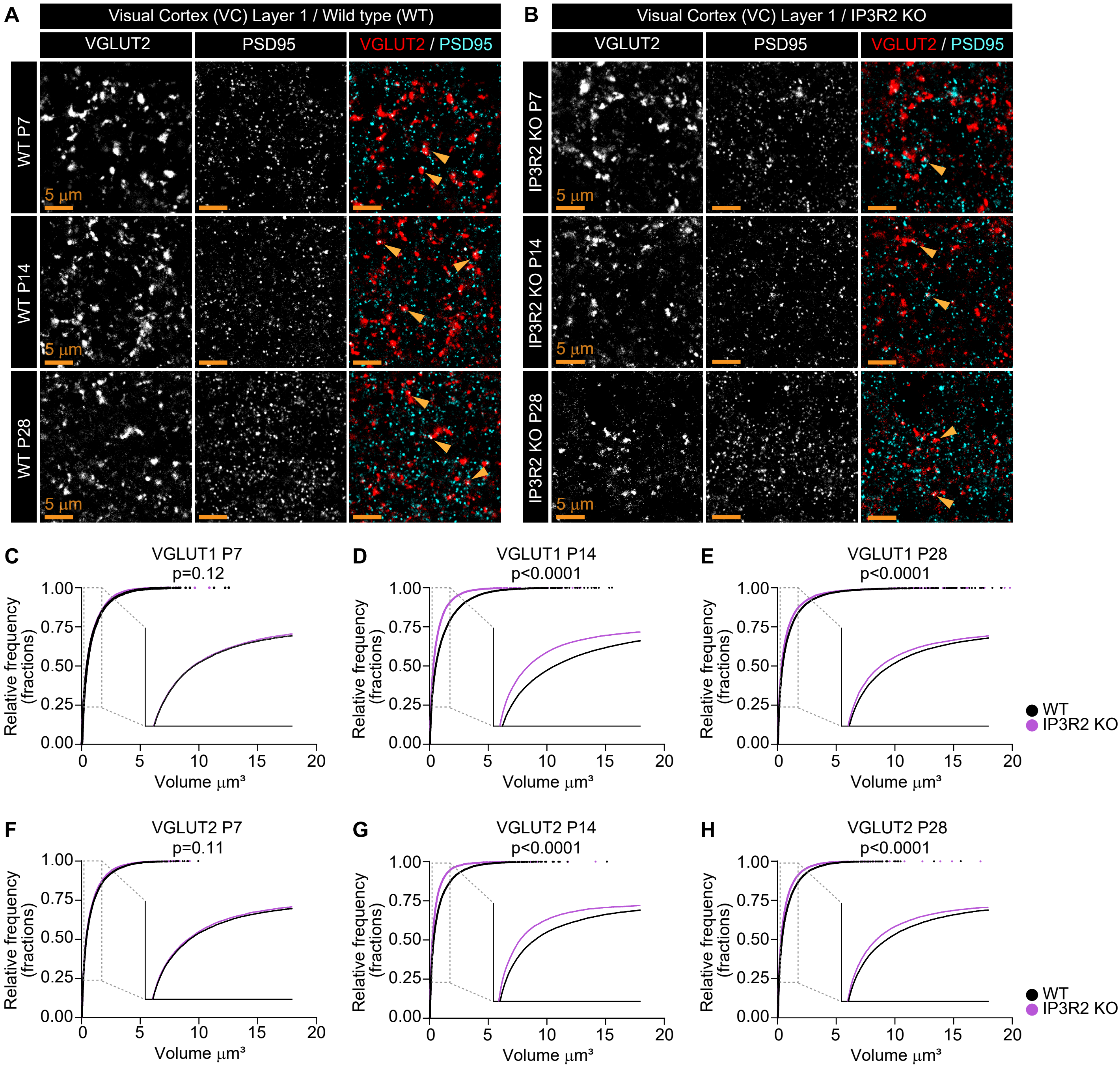

A
Visual Cortex (VC) Layer 1 / Wild type (WT)
VGLUT2
PSD95
VGLUT2 / PSD95
WT P7
WT P14
WT P28
5 µm
B
Visual Cortex (VC) Layer 1 / IP3R2 KO
VGLUT2
PSD95
VGLUT2 / PSD95
IP3R2 KO P7
IP3R2 KO P14
IP3R2 KO P28
5 µm
C
VGLUT1 P7
p=0.12
D
VGLUT1 P14
p<0.0001
E
VGLUT1 P28
p<0.0001
F
VGLUT2 P7
p=0.11
G
VGLUT2 P14
p<0.0001
H
VGLUT2 P28
p<0.0001
Relative frequency (fractions)
Volume µm³
WT
IP3R2 KO

Figure S2. VGLUT volumes are reduced in IP3R2 KO mice; related to Figure 1. See also Supplementary Data 1. **A-B.** Example images of the presynaptic VGLUT2, postsynaptic PSD95 and merged (synapses) in each age and genotype as labeled. Single channel grayscale images on the left, merged images on the right. **C-H.** Cumulative distributions of volumes from 3D Imaris rendered images (see Fig. 1, Fig. S1) for VGLUT1 (C-E) and VGLUT2 (F-H) per age and genotype as labeled. Insets show enlarged data from dashed outlined box. Higher fraction of smaller volumes (<5 μm^3) is observed in IP3R2 KO mice at P14 and P28, but not P7. P value by Kolmogorov-Smirnov test comparing WT and KO within each age is noted on each plot.

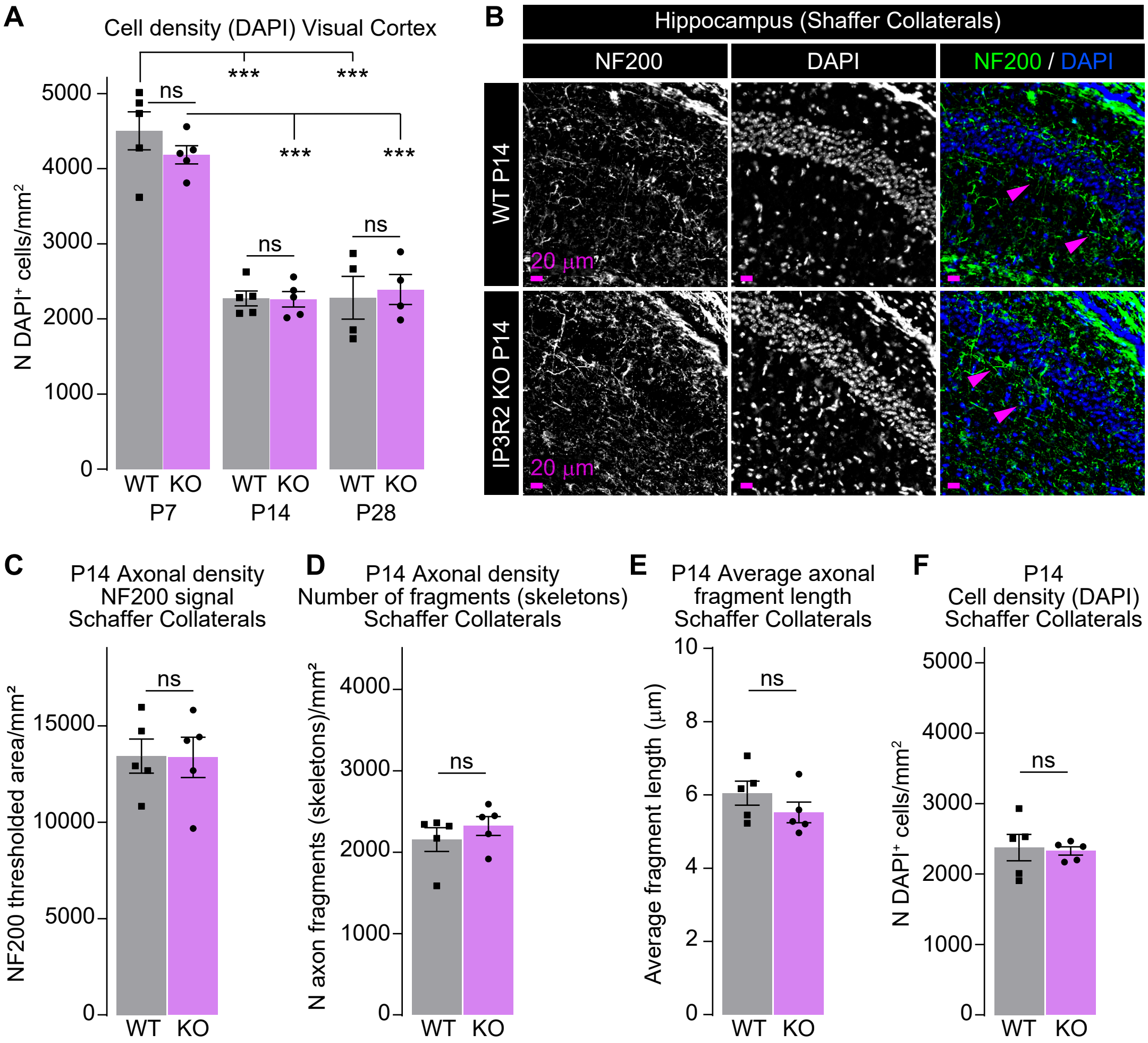

A
Cell density (DAPI) Visual Cortex
ns
***
***
ns
ns
N DAPI+ cells/mm2
5000
4000
3000
2000
1000
0
WT KO WT KO WT KO
P7 P14 P28
B
Hippocampus (Shaffer Collaterals)
NF200
DAPI
NF200 / DAPI
WT P14
IP3R2 KO P14
20 µm
20 µm
C
P14 Axonal density
NF200 signal
Schaffer Collaterals
NF200 thresholded area/mm2
15000
10000
5000
0
ns
WT KO
D
P14 Axonal density
Number of fragments (skeletons)
Schaffer Collaterals
N axon fragments (skeletons)/mm2
4000
3000
2000
1000
0
ns
WT KO
E
P14 Average axonal
fragment length
Schaffer Collaterals
Average fragment length (µm)
10
8
6
4
2
0
ns
WT KO
F
P14
Cell density (DAPI)
Schaffer Collaterals
N DAPI+ cells/mm2
5000
4000
3000
2000
1000
0
ns
WT KO

Figure S3. Gross axonal density and cell numbers are unchanged in IP3R2 KO mice; related to Figure 2. See also Supplementary Data 1. **A.** Quantification of Fig. 2A-B showing cell density (represented by number of DAPI positive ($^+$) cells normalized to total area in mm^2) is decreased across development from P7 to P28 similarly in WT and KO groups, with no difference between genotypes at any age tested. **B-F.** Hippocampal Schaffer collateral axon density and cell numbers are similar at P14 in WT and IP3R2 KO mice. **B.** Representative images of hippocampus in WT (top) and IP3R2 KO (bottom) labeled for NF200 and DAPI. Arrowheads indicate representative Schaffer collateral axons. **C-F.** Quantification of B showing NF200 fluorescent signal (C), number of skeletonized axonal fragments (D), average axonal fragment length (from skeletonize-based analysis as in C, Fig. 2D-E) (E), and DAPI (F) cell density. Graphs show mean ± s.e.m.; individual mouse data points are shown as circles and squares. Number of mice/ group (N) N=5. Statistical analysis in A, by two-way ANOVA with multiple comparison correction by the two-stage linear step-up procedure of Benjamini, Kriger and Yekutieli examining the effects of age and genotype; *P≤0.05; **P<0.01; ***P<0.001 comparing age groups within each genotype; P value (p) on the plots comparing WT and KO groups. In C-F, statistical analysis by t-test. ns denotes non-significant (P>0.05) results. Descriptive statistics and detailed statistical test results for all data panels are provided in Supplementary Data 1.

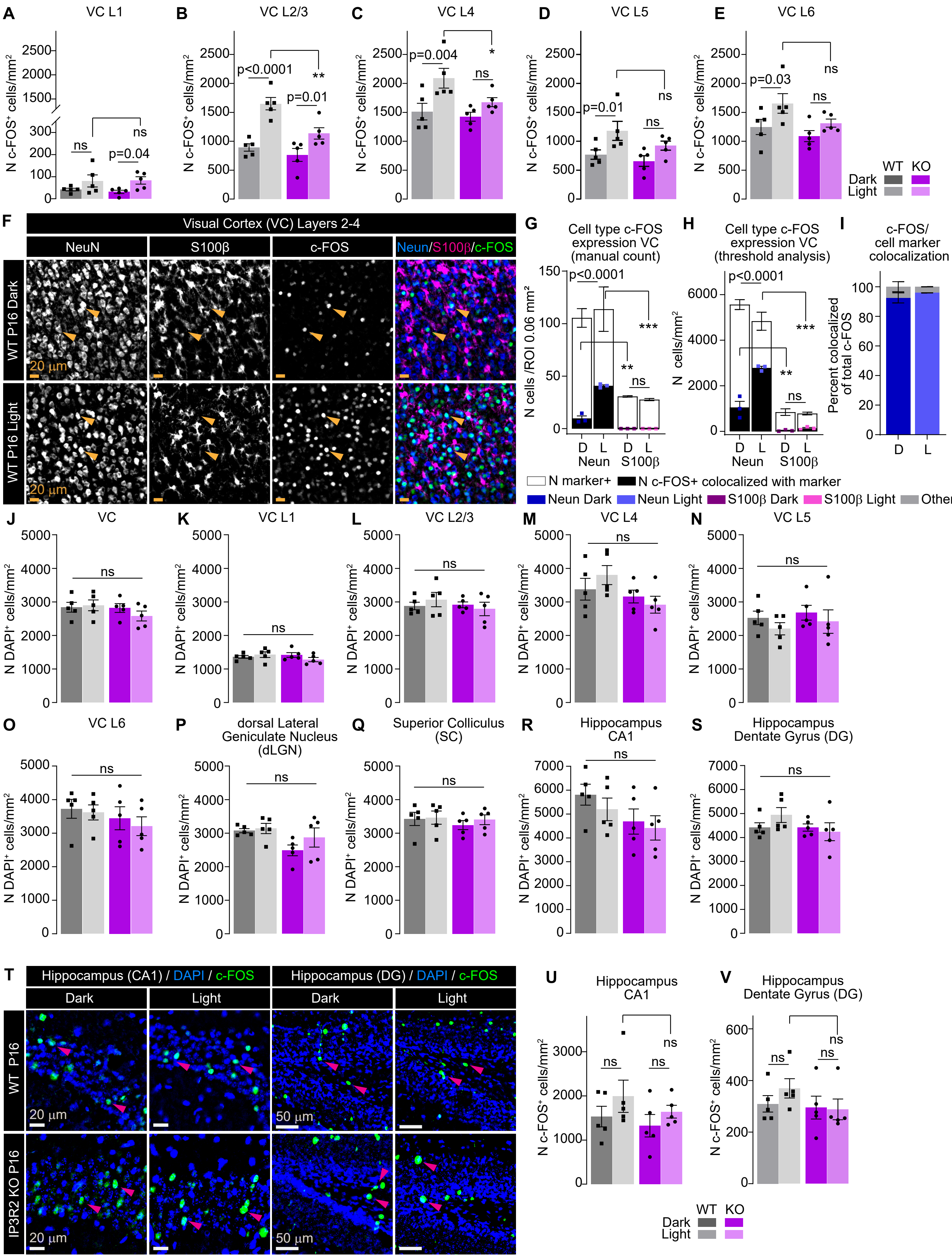
A VC L1
B VC L2/3
C VC L4
D VC L5
E VC L6
N c-FOS+ cells/mm2
p<0.0001
p=0.004
p=0.01
p=0.03
p=0.04
ns
WT KO
Dark
Light
F Visual Cortex (VC) Layers 2-4
NeuN
S100β
c-FOS
Neun/S100β/c-FOS
WT P16 Dark
WT P16 Light
20 μm
G Cell type c-FOS expression VC (manual count)
N cells /ROI 0.06 mm2
H Cell type c-FOS expression VC (threshold analysis)
N cells/mm2
I c-FOS/ cell marker colocalization
Percent colocalized of total c-FOS
D L D L
Neun S100β
N marker+
N c-FOS+ colocalized with marker
Neun Dark
Neun Light
S100β Dark
S100β Light
Other
J VC
K VC L1
L VC L2/3
M VC L4
N VC L5
O VC L6
P dorsal Lateral Geniculate Nucleus (dLGN)
Q Superior Colliculus (SC)
R Hippocampus CA1
S Hippocampus Dentate Gyrus (DG)
N DAPI+ cells/mm2
T Hippocampus (CA1) / DAPI / c-FOS
Hippocampus (DG) / DAPI / c-FOS
Dark
Light
WT P16
IP3R2 KO P16
50 μm
U Hippocampus CA1
V Hippocampus Dentate Gyrus (DG)

Figure S4. Light-evoked c-FOS expression is blunted in IP3R2 KO mice visual circuit; related to Figure 4. See also Supplementary Data 1. **A-E.** Quantification of Fig. 4C separated by cortical layers represented as c-FOS positive cell numbers per area in mm^2 (c-FOS cell density). Light exposure induces a significant increase in c-FOS positive cell density in the WT in layers (L) 2/3, 4, 5, and 6, and significant increase in L1 and 2/3 in the KO group. Light-evoked c-FOS density is significantly lower in the KO compared to WT in layers 2/3 and 4. **F-I.** c-FOS expression is restricted to neurons at P16 with minimal expression in astrocytes or other cell types in both dark and light conditions. **F.** Example images of the VC L2-4 region showing c-FOS (green) astrocyte marker S100β (magenta), and neuronal marker Neun (blue) in dark and light exposed WT mice as labeled. **G-H.** Quantification of F performed by either manual count of c-FOS positive cells expressing cell type markers (G), or semi-automated thresholding analysis of larger cortical area (H). Open bars represent the total numbers of Neun and S100β positive cells, while black bars within the open bars represent c-FOS positive cells that co-expressed each of the cell type markers as labeled. Significantly higher colocalization of c-FOS positive cells with Neun compared to S100β is observed. **I.** Analysis of data in G showing percentage of total c-FOS positive cells expressing each cell type marker (Neun for neurons, S100β for astrocytes, or Other for neither cell type) showing ~95% of c-FOS expressing cells positive for Neun with no S100β positive c-FOS expressing cells detected. **J-S.** Quantification of DAPI positive cells shows no difference between genotypes or light conditions in any of the analyzed brain regions as labeled. **T-V.** Light exposure does not induce increased c-FOS expression in the hippocampus. Example images of c-FOS (green) and DAPI to mark cell nuclei (blue) in the CA1 and DG regions of the hippocampus in both genotypes as labeled (T), as well as quantification for CA1 (U) and dentate gyrus (DG) (V) are shown. No difference is observed between light and dark conditions or the genotypes in either sub-region. Graphs show mean ± s.e.m.; individual mouse data points are shown as circles and squares. Number of mice/group (N) N=5, KO DG N=4. Scale bar VC, CA1 = 20 μm, DG = 50 μm. Arrowheads mark representative c-FOS positive ($^+$) cells. Statistical analysis by two-way ANOVA with multiple comparison correction by the two-stage linear step-up procedure of Benjamini, Kriger and Yekutieli examining the effects of light exposure and genotype; $^*P\leq0.05$; $^{**}P<0.01$; $^{***}P<0.001$ comparing the effect of light pulse within each genotype; P value (p) on the plots comparing WT and KO groups. ns denotes non-significant results ($P>0.05$). Descriptive statistics and detailed statistical test results for all data panels are provided in Supplementary Data 1.

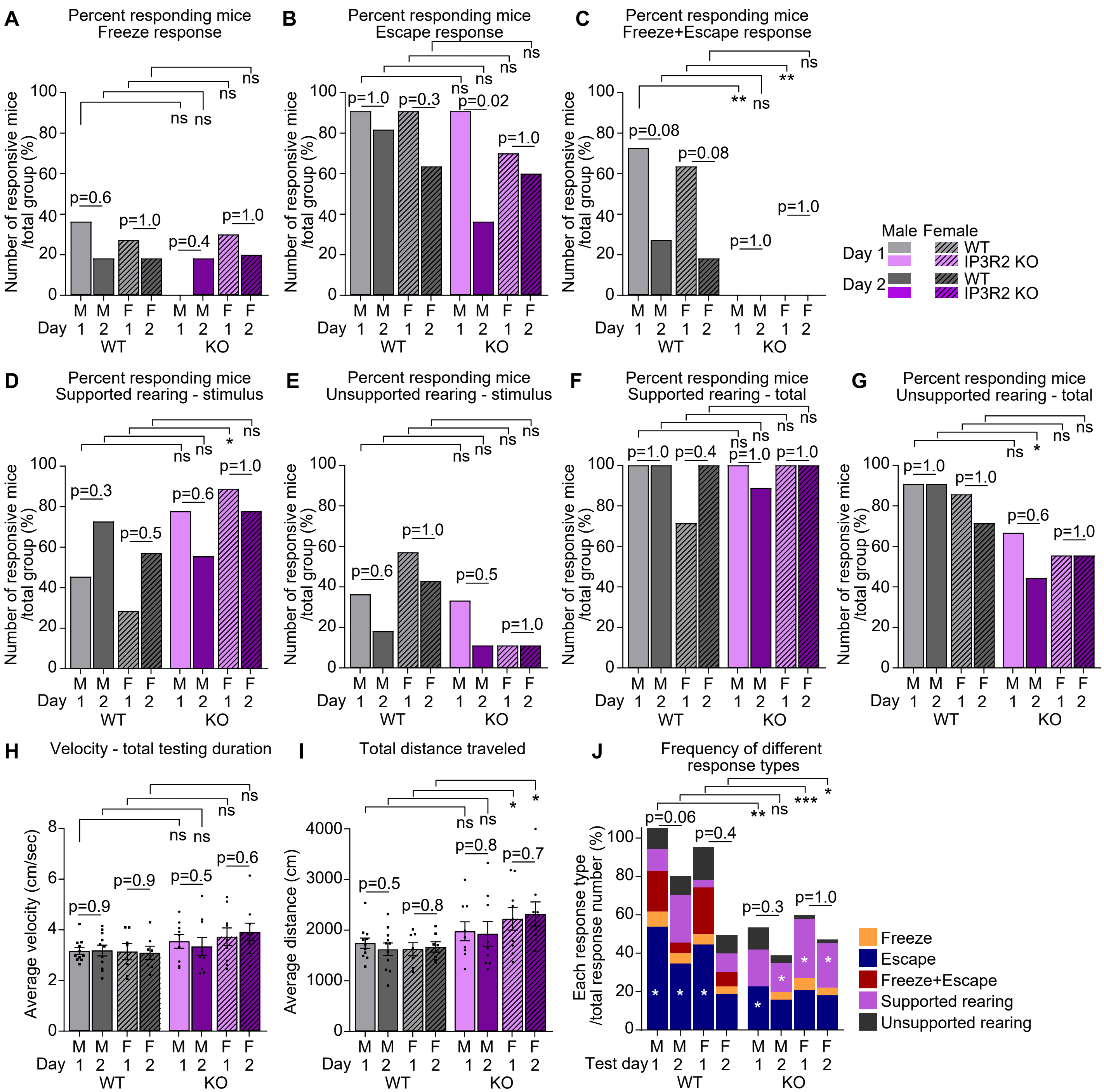
A
Percent responding mice
Freeze response
B
Percent responding mice
Escape response
C
Percent responding mice
Freeze+Escape response
Number of responsive mice
/total group (%)
p=0.6
p=1.0
p=0.4
p=1.0
p=1.0
p=0.3
p=0.02
p=0.08
p=0.08
ns
**
Day
WT
KO
Male Female
Day 1
Day 2
WT
IP3R2 KO
D
Percent responding mice
Supported rearing - stimulus
E
Percent responding mice
Unsupported rearing - stimulus
F
Percent responding mice
Supported rearing - total
G
Percent responding mice
Unsupported rearing - total
p=0.5
p=0.6
p=0.4
*
H
Velocity - total testing duration
Average velocity (cm/sec)
p=0.9
p=0.5
I
Total distance traveled
Average distance (cm)
p=0.8
p=0.7
J
Frequency of different
response types
Each response type
/total response number (%)
p=0.06
***
Test day
Freeze
Escape
Freeze+Escape
Supported rearing
Unsupported rearing

Figure S5. Visually evoked defensive responses and rearing behaviors are disrupted in IP3R2 KO mice; related to Figure 5. See also Supplementary Data 1. **A-E.** Number of responders to the looming stimulus represented as percentage of mice responding to at least one trial per day out of total group number for each of the response types as labeled. All groups exhibited a similar percentage of responders, except for the "Freeze+Escape" response, which was absent in KO mice (C), and "Supported rearing" response, which has higher percentage of responding KO females compared to WT (statistically significant on Day 1) (D). **F-G.** Number of mice exhibiting the rearing behavior (supported – F; unsupported – G) during the total testing period represented as percentage of mice showing at least one rearing bout per testing day out of total group number. Almost all the mice exhibited at least one "Supported rearing" response on both testing days. Percentage of mice exhibiting the "Unsupported rearing" behavior is lower in KO compared to WT (significant in males Day 2), with no difference between testing days. **H-I.** Velocity (H) and total distance traveled (I) averaged across the total testing period, showing similar locomotion between testing days for each sex, with a significant increased total distance traveled in KO females compared to WT females. **J.** Fractions of each of the defensive response types to the looming stimulus ("Freeze" – yellow; "Escape" – blue; "Freeze+Escape" – maroon; "Supported rearing" – purple; "Unsupported rearing" – dark gray) out of total responses shown as percentage for each age and sex as labeled. Supported/Unsupported rearing events can co-occur with other response types resulting in higher than 100% total responses. Number of mice (N) N=7-11/sex/genotype. Statistical analysis in A-G, J by Fisher's exact test with Monte Carlo simulation for large arrays, in H-I by two-way ANOVA with multiple comparison correction by the two-stage linear step-up procedure of Benjamini, Kriger and Yekutieli examining the effects of testing day and genotype. $^*P \leq 0.05$; $^{**}P<0.01$; $^{***}P<0.001$ comparing between WT and KO groups within each sex; P value (p) on the plots comparing Day 1 and Day 2 responses within each sex and genotype. In J, asterisk within each bar marks the most frequent response type. ns denotes non-significant results ($P>0.05$). Descriptive statistics and detailed statistical test results for all data panels are provided in Supplementary Data 1.

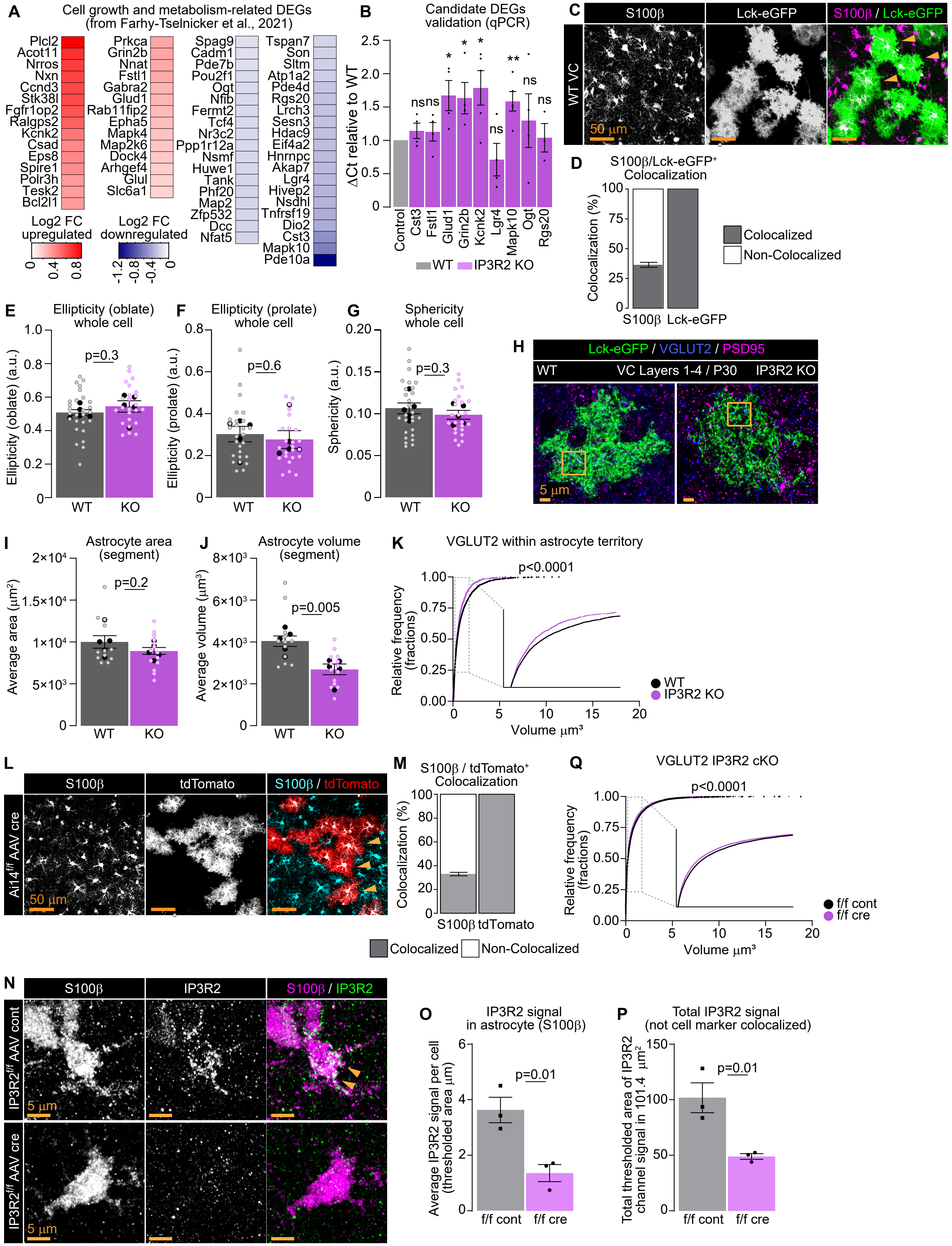
A
Cell growth and metabolism-related DEGs (from Farhy-Tselnicker et al., 2021)
Plcl2 Acot11 Nrros Nxn Ccnd3 Stk38l Fgfr1op2 Ralgps2 Kcnk2 Csad Eps8 Spire1 Polr3h Tesk2 Bcl2l1
Prkca Grin2b Nnat Fstl1 Gabra2 Glud1 Rab11fip2 Epha5 Mapk4 Map2k6 Dock4 Arhgef4 Glul Slc6a1
Spag9 Cadm1 Pde7b Pou2f1 Ogt Nfib Fermt2 Tcf4 Nr3c2 Ppp1r12a Nsmf Huwe1 Tank Phf20 Map2 Zfp532 Dcc Nfat5
Tspan7 Son Sltm Atp1a2 Pde4d Rgs20 Lrch3 Sesn3 Hdac9 Eif4a2 Hnrnpc Akap7 Lgr4 Hivep2 Nsdhl Tnfrsf19 Dio2 Cst3 Mapk10 Pde10a
Log2 FC upregulated
Log2 FC downregulated
B
Candidate DEGs validation (qPCR)
ΔCt relative to WT
Control Cst3 Fstl1 Glud1 Grin2b Kcnk2 Lgr4 Mapk10 Ogt Rgs20
WT IP3R2 KO
C
S100β Lck-eGFP S100β / Lck-eGFP
WT VC
50 μm
D
S100β/Lck-eGFP+ Colocalization
Colocalization (%)
Colocalized
Non-Colocalized
S100β Lck-eGFP
E
Ellipticity (oblate) whole cell
p=0.3
F
Ellipticity (prolate) whole cell
p=0.6
G
Sphericity whole cell
p=0.3
WT KO
H
Lck-eGFP / VGLUT2 / PSD95
WT VC Layers 1-4 / P30 IP3R2 KO
5 μm
I
Astrocyte area (segment)
p=0.2
Average area (μm²)
J
Astrocyte volume (segment)
p=0.005
Average volume (μm³)
K
VGLUT2 within astrocyte territory
p<0.0001
Relative frequency (fractions)
Volume μm³
WT
IP3R2 KO
L
S100β tdTomato S100β / tdTomato
Ai14f/f AAV cre
50 μm
M
S100β / tdTomato+ Colocalization
Colocalization (%)
S100β tdTomato
Colocalized Non-Colocalized
Q
VGLUT2 IP3R2 cKO
p<0.0001
f/f cont
f/f cre
N
S100β IP3R2 S100β / IP3R2
IP3R2f/f AAV cont
IP3R2f/f AAV cre
5 μm
O
IP3R2 signal in astrocyte (S100β)
Average IP3R2 signal per cell (thresholded area μm)
p=0.01
P
Total IP3R2 signal (not cell marker colocalized)
Total thresholded area of IP3R2 channel signal in 101.4 μm²
p=0.01
f/f cont f/f cre

Figure S6. Reduced astrocytic morphogenesis and synapse contact germline IP3R2 KO mice, and reduced synapse numbers in astrocyte-specific conditional IP3R2 cKO VC; related to Figure 6. See also Supplementary Data 1. **A.** Heatmap showing Log2 fold change for cell growth and metabolism-related differentially expressed genes (DEGs; obtained from published single nucleus RNA sequencing dataset, Farhy-Tselnicker et al., 2021[4]) in IP3R2 KO mice relative to WT. **B.** Quantification of qPCR represented as change in IP3R2 KO Ct relative to *Gapdh* (ΔCT) normalized to WT. Number of mice/group (N) N=3-5 per gene, each reaction run in triplicates. *P≤0.05; **P<0.01; ns denotes non-significant results (P>0.05) by t-test. **C-D.** Validation of AAV expression showing specificity and sparsity of Lck-eGFP labeling in VC astrocytes. Example images showing Lck-eGFP (green) and astrocyte marker S100β (magenta) (C) and quantification (D). Plot shows percentage of double labeled cells out of total astrocytes (S100β positive) or Lck-eGFP positive showing full overlap of Lck-eGFP expressing cells with S100β. **E-G.** Astrocytic shape characteristics including ellipticity and sphericity are unaltered by IP3R2 KO. **H-K.** Astrocyte volume, and synapse numbers within astrocyte territories are reduced in IP3R2 KO mice at P30. **H.** Example images of astrocytes from P30 mice VC expressing Lck-eGFP (green) co-labeled with synaptic markers VGLUT2 (blue) and PSD95 (magenta) for each genotype as labeled. Boxes are zoomed-in regions depicted in Fig. 6F. **I-J.** Quantification of astrocyte morphological parameters showing no change in area (I), and significant reduction in volume (J) in IP3R2 KO astrocytes. **K.** Cumulative distribution of VGLUT2 volumes from 3D Imaris rendered images (Fig. 6G) per genotype as labeled. Higher fraction of smaller volumes (<5 μm^3) is observed within the territories of IP3R2 KO astrocytes. **L-M.** Validation of ICV injected AAV-mediated cre recombinase expression efficiency and astrocyte specificity using cre-dependent tdTomato reporter mouse line (Ai14, see Methods). Example images showing tdTomato reporter expression (red) and astrocyte marker S100β (cyan) (L) and quantification plot showing percentage of double labeled cells (GFP and S100β) out of total astrocytes (S100β positive) showing ~30% recombination efficiency, and 100% overlap of tdTomato expressing cells with S100β confirming astrocyte specificity (M). **N-P.** Validation of postnatal astrocyte specific knockout of IP3R2 at P16 following AAV injection into $IP3R2^{fl/fl}$ mice at P1-2 by IHC. **N.** Example images showing IP3R2 signal (green) and astrocyte marker S100β (magenta) in control and cre AAV injected groups as labeled. Single channel grayscale images on the left, merged image on the right. **O-P.** Quantification of N, showing reduced IP3R2 signal colocalized with S100β (O) and total quantification of IP3R2 channel (P) (non-colocalized, as in Fig. S1C) in cre expressing group. **Q.** Cumulative distribution of VGLUT2 volumes from 3D Imaris rendered images (Fig. 6L) per group as labeled. Significant increase in fraction of smaller volumes (<5 μm^3) is observed in cKO group.

Bar graphs show mean ± s.e.m. Black circles or squares are average of signal in each individual mouse; colored open circles are data for each astrocyte in E-G, I-J. Number of mice/group (N) N=5, in N-O = 4; number of astrocytes = 25-27. Scale bar in C, L = 50 μm; in H, N = 5 μm. P value (p) indicated on each plot, by t-test; in K, Q, P value (p) indicated on each plot by Kolmogorov-Smirnov test. Descriptive statistics and detailed statistical test results for all data panels are provided in Supplementary Data 1.
