# Supplementary material for: Astrocyte store released calcium modulates structural synapse development and circuit output in the visual system
Source: Commun Biol. 2026 Jul 11;9:1456. doi: 10.1038/s42003-026-10579-0 (PMC13737306; doi:10.1038/s42003-026-10579-0)
Supplement: Supplementary file 2 [file 42003_2026_10579_MOESM2_ESM.pdf]

Description of Additional Supplementary Files

File name: Supplementary Data 1

Description: source data behind the graphs in the paper and statistical analysis results for all data.
